# Enhancement of hidden Markov model analyses for improved inference of archaic introgression in modern humans

**DOI:** 10.1093/molbev/msag134

**Published:** 2026-06-03

**Authors:** Moisès Coll Macià, Laurits Skov, Zenia Elise Damgaard Bæk, Asger Hobolth

**Affiliations:** Bioinformatics Research Centre, Aarhus University, Aarhus C, Denmark; Section for Molecular Ecology and Evolution, Globe Institute, University of Copenhagen, Copenhagen, Denmark; National Centre for Register-Based Research, Department of Public Health, Aarhus University, Aarhus C, Denmark; Department of Mathematics, Aarhus University, Aarhus C, Denmark

**Keywords:** hidden Markov models, inhomogeneous Markov chain, sampling from the posterior, Finite Markov Chain Imbeding, hybrid decoding, hmmix, archaic introgression

## Abstract

Insights into the admixture history between modern and archaic humans require accurately inferred introgressed fragments within modern genomes. Here, we introduce two enhancements to hidden Markov models (HMMs) implemented in hmmix. First, we develop a method for sampling hidden state sequences conditional on observed genomic data, enabling robust estimation of admixture summary statistics—such as admixture proportion and fragment length distributions. This represents an improvement compared to relying solely on point estimates as provided by classical decoding methods. Additionally, we integrate the Finite Markov Chain Imbedding (FMCI) framework, allowing exact analytical calculation of these admixture statistics, tailored to large scale human genomes. Second, we implement a novel hybrid decoding method which combines the strengths of Viterbi and Posterior decoding methods, substantially improving the reliability of archaic fragments identified. We validate these improvements on data from the 1000 Genomes Project and demonstrate that our sampling method yields more accurate admixture estimates from single individuals compared to existing approaches requiring extensive population-level datasets. Moreover, we show how hybrid decoding can be instrumental in resolving the inference of local archaic haplotype structure in modern human genomes. These methodological advancements will enhance HMM-based analyses in any field of science and will provide deeper insight into the complex history of genetic interactions between archaic and modern human populations.

## Introduction

Decoding archaic content in modern humans provides insights into past interactions between our ancestors and the extinct Neanderthals and Denisovans (Sankararaman et al. 2016; Bergström et al. 2020; Skov et al. 2020; Iasi et al. 2024). A key tool in this effort is hmmix (Skov et al. 2018), which identifies archaic fragments in human genomes based on Hidden Markov Models (HMMs). HMMs are well-suited for this task due to the sequential nature of genomic data and because archaic ancestry cannot be directly measured, but must be inferred from indirect genetic signatures.

In general, Posterior decoding and Viterbi decoding are the main methods to decode the hidden state sequences using HMMs (Zucchini et al. 2017). For the case of hmmix, Posterior decoding is the default option as its results are more accurate due to its definition of optimizing local probabilities by maximizing accuracy in each position of the sequence. In contrast, Viterbi decoding chooses the hidden path with the highest probability among all possible hidden paths conditional on the observed sequence. This approach optimizes a global property and the resulting hidden path has substantially fewer hidden state transitions than Posterior decoding. Although this behavior reduces its risk for detecting false archaic fragments (false positives), it makes the model miss short yet genuine archaic fragments.

Despite their respective strengths, each decoding method represents extreme optimization strategies, either local or global, without possible intermediate solutions. Another limitation of the existing decoding methods is that both produce deterministic outputs, yielding a single fixed set of archaic fragments per genome, without an explicit quantification of uncertainty. This lack of uncertainty estimation can bias downstream analyses based on summary statistics, such as estimates of admixture proportions, potentially leading to inaccurate interpretations of archaic ancestry in the genome analyzed.

To address these issues, we describe two key enhancements for general HMM frameworks in a separate manuscript (Bæk et al. 2025), and here tailored to hmmix. First, we use the fact that the hidden state sequence, conditional on the observed sequence, is an inhomogeneous Markov chain. The inhomogeneous transition probabilities along the sequence can be found from the backward table of the HMM and that enables the estimation of the full distributions of the summary statistics of interest, with a sampling based as well as an analytical based approach (Aston and Martin 2007). From this, we obtain a more accurate and nuanced estimation of key insights in the study of archaic admixture when data is consistent with the hmmix model and parameters are correctly inferred. Second, we implement the hybrid decoding method (Lember and Koloydenko 2014; Kuljus and Lember 2023). This method estimates hidden state sequences that are intermediate solutions between Posterior and Viterbi outputs by combining the strengths of both global and local decoding optimizations, respectively. Finally, we check robustness of these methodological enhancements with coalescent based simulations and apply these to three real phased genomes. We demonstrate that the new methodologies allow us to obtain more refined insights about the admixture history between modern and archaic humans, showcasing the improvements that can be obtained when applying these approaches to HMM analyses.

## Results

### Hmmix

**Figure 1:**
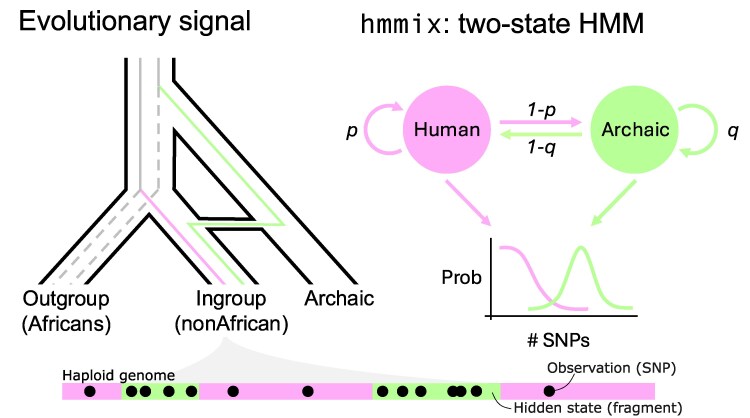
Graphical representation of the evolutionary signal (left) that hmmix uses as input and the model itself (right). The tree shows the phylogenetic relationship between the outgroup, ingroup and the archaic group. Only the ingroup receives gene-flow from the archaic group. The lines within the tree represent archaic and modern human lineages within the ingroup genome. Colorful parts of the lineages show the time while each lineage accumulates private genetic variation compared to the outgroup. A representation of a haploid genome from an ingroup individual is represented as a strip with human and archaic fragments. Black dots represent private derived variation accumulated in those regions, with archaic fragments being more dense than human fragments. The graph on the right represents hmmix as the two-state HMM with their transition probabilities between archaic and human states and their corresponding emission probabilities with different Poisson rates depending on SNP density.

Non-African human genomes carry ∼2% to 5% Neanderthal and Denisovan DNA, a legacy from the encounters between archaic hominins and early modern humans during their expansion out of Africa (Bergström et al. 2020). As a result, non-Africans have accumulated derived genetic variants that are private when compared to their African counterparts in two distinct ways: through new mutations that arose after the divergence from African groups, and through archaic admixture. Notably, archaic fragments in modern human genomes exhibit a much higher density of private and derived variants, proportional to the longer evolutionary timespan to accumulate those (Fig. 1).

The hmmix software (Skov et al. 2018) leverages this contrast of private SNP density to identify genomic regions of archaic origin in test genomes (Fig. 1). It employs a two-state HMM in which the hidden states represent archaic and human genetic origin. Observed SNP densities in 1,000 bp (1 kb) windows are modeled as Poisson-distributed, with different rates based on the underlying hidden state. To achieve this difference of SNP density in genomic data, shared variants with a set of African genomes (outgroup) are excluded from the test genome (ingroup). Compared to other methods (Sankararaman et al. 2014; Chen et al. 2020), this design enables hmmix to infer archaic fragments without requiring a reference archaic genome, thereby reducing potential bias toward any specific sequenced Neanderthal or Denisovan genome. Nonetheless, sequenced archaic genomes are usually used downstream to annotate and classify identified archaic fragments.

To evaluate the advances implemented in hmmix, we simulate archaic fragments from a two-state HMM—the same as the hmmix model—under two distinct parameter sets with hmmix (Materials and methods). The first set, referred to as realistic parameters, mimics the Neanderthal admixture scenario and is used for the statistical evaluation of the different methods presented (Table 1). The second parameter set, named as visualization parameters, produces a much higher overall archaic content and is designed mainly for visualization purposes and facilitating interpretation (Table 2).

**Table 1:** Realistic parameters and their Baum-Welch estimated parameters with their 95% CI (Materials and methods).

Realistic parameters				
	Emission probabilities (*λ*)	Starting probabilities (*π*)	Transition probabilities (*Γ*)	
			Human	Archaic
Human	0.03	0.98	0.9995918	0.0004082
Archaic	0.3	0.02	0.02	0.98

Baum-Welch estimated realistic parameters				
	Emission probabilities (*λ*)	Starting probabilities (*π*)	Transition probabilities (*Γ*)	
			Human	Archaic
Human	0.0300 (0.0299 to 0.0302)	0.9808 (0.9796 to 0.9820)	0.9996 (0.9996 to 0.9996)	0.0004 (0.0004 to 0.0004)
Archaic	0.3039 (0.3000 to 0.3090)	0.0192 (0.0180 to 0.0204)	0.0204 (0.0191 to 0.0215)	0.9796 (0.9785 to 0.9809)

The estimated parameters are rounded to the fourth digit.

**Table 2:** Visualization parameters and their Baum-Welch estimated parameters.

Visualization parameters				
	Emission probabilities (*λ*)	Starting probabilities (*π*)	Transition probabilities (*Γ*)	
			Human	Archaic
Human	0.2	0.9	0.99	0.01
Archaic	0.5	0.1	0.02	0.98

Baum-Welch estimated visualization parameters				
	Emission probabilities (*λ*)	Starting probabilities (*π*)	Transition probabilities (*Γ*)	
			Human	Archaic
Human	0.200	0.674	0.990	0.010
Archaic	0.500	0.327	0.020	0.980

### Summary distributions from the conditional hidden state sequence

The hidden state sequence in a HMM, conditioned on the observed data, constitutes an inhomogeneous Markov chain. In this case, the transition probabilities between the hidden states become inhomogeneous along the sequence (Materials and methods, Bæk et al. 2025). In other words, the probability of staying in the same state or changing to another state varies throughout the sequence depending on the observations. Aston and Martin discussed this property, highlighting its potential for sampling from the conditional distribution of state sequences (Aston and Martin 2007). Thus, we can generate a collection of simulated hidden state sequences with this approach, reflecting the variance in the conditional posterior probabilities.

**Figure 2:**
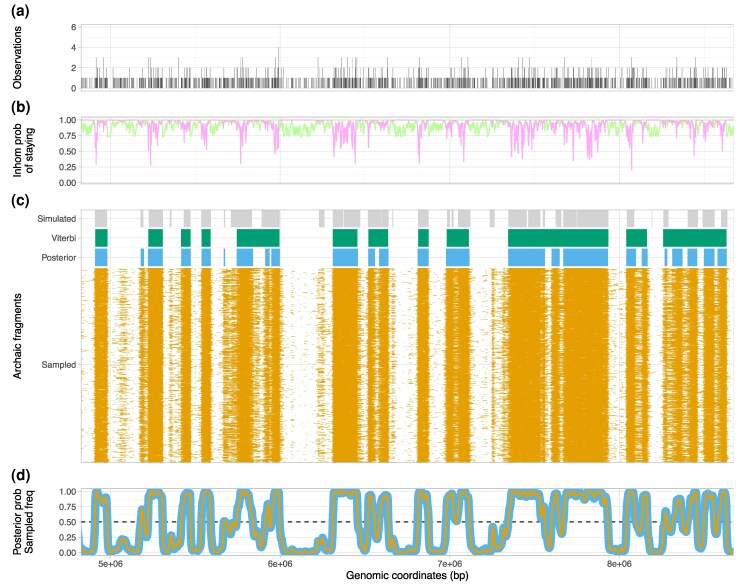
Simulated genetic region analysis from visualization parameters with results from different decoding methods. a) The observations as counts of SNPs in 1,000 bp windows as the input for hmmix. b) Inhomogeneous transition probabilities of staying in the same state (Archaic $b_{t}$ in green and Human $a_{t}$ in pink) conditional on the observed data. c) The archaic fragments from true simulated, Viterbi decoded, Posterior decoded and 1,000 samples from the conditional distribution of hidden states. d) The posterior probability of being archaic from Posterior decoding (blue) and the frequency of archaic sequence in the 1,000 sampled paths (orange). Note that the two lines are superposed and that differences cannot be visually distinguished. The dashed black line denotes the posterior probability threshold (50%) to decide if a window is considered archaic or human for Posterior decoding.

Building on this idea, we implement in hmmix a procedure for sampling from the inhomogeneous Markov chain of hidden state sequences conditional on the observed data (Materials and methods). In Fig. 2a we show a simulated observed sequence from the visualization parameters (Materials and methods), and in Fig. 2b we show the corresponding inhomogeneous transition probabilities of staying in each state conditional on the observed sequence (Materials and methods). The probabilities of staying in the archaic state $b_{t}$ increase with the number of observed variants per window. Conversely, the probabilities of staying in the human state $a_{t}$ decrease in the same regions. The reverse is true for low-density regions. This behavior aligns with the design of hmmix, where archaic regions are characterized by high variant density, while human regions correspond to lower densities (Fig. 1).

Using the inhomogeneous transition probabilities, we sample 1,000 hidden state sequences for the visualization parameters’ simulated data with hmmix (Materials and methods). These sequences are compared against the true simulated sequences, as well as those inferred by Viterbi and Posterior decoding implemented also in hmmix (Fig. 2c).

Occasionally, sampling from the posterior is able to capture fragments missed by both Viterbi and Posterior decoding, proportional to their posterior probability (Fig. 2c). In the case of Posterior decoding, these fragments are missed because only fragments with posterior probabilities above 50% of being archaic are classified as such (Fig. 2d). In fact, the frequency of a given position to be classified as archaic among all samples corresponds to its posterior probability as estimated by Posterior decoding (Pearson correlation test = 0.9997, *P* value < 2.2 × 10^−16^, Fig. 2d).

**Figure 3:**
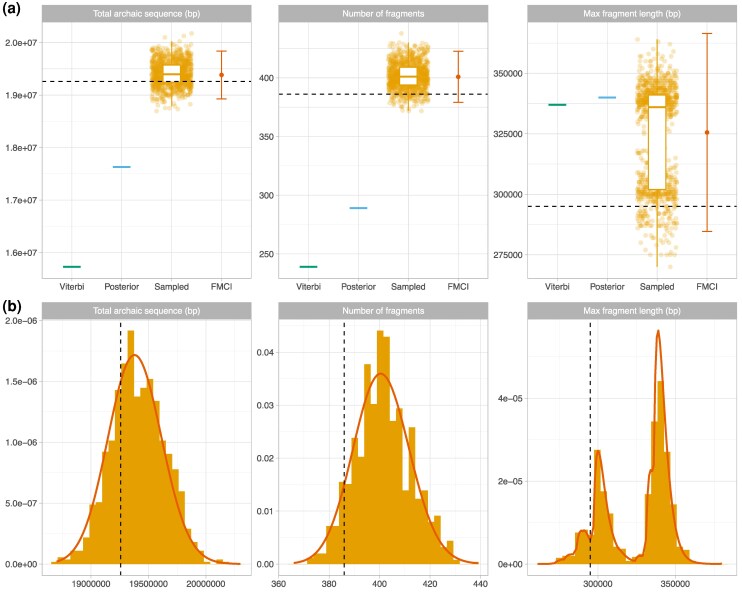
Summary statistics of archaic fragments based on realistic parameter simulations. a) Comparison between the true value form simulations (dash lines) with the summary obtained from each decoding method. The Viterbi and Posterior decoding summary values are shown as horizontal lines. Individual values from each path sampled from the posterior (1,000 paths) are shown as orange dots and the distribution of all values are summarized with a box plot. The mean of the FMCI distribution is shown as a dark orange dot with the 1.96 standard deviation around the mean as error bars. b) Distribution of summary statistics from 1,000 paths sampled from the posterior (histogram) and the analytical FMCI distribution (line).

The sampling approach allows us to compute the distribution of classical summary statistics of interest in the study of archaic admixture, such as total amount of archaic sequence, archaic fragments length distribution, total number of archaic fragments and the longest archaic fragment. These statistics are relevant as, for example, the total amount of archaic sequence in a sampled human genome is often used to estimate the admixture proportion from Neanderthals. Figure 3a shows three of the summary statistics obtained with the different decoding methods presented above and compares them to the true simulated value from realistic parameters. While only point estimates are obtained with the two classical decoding methods, with the sampling approach we are able to quantify the variance around the estimated summary of interest. For the number of archaic fragments and amount of archaic sequence, the true value is captured by the distribution of summaries obtained with the 1,000 sampled paths. Conversely, both Viterbi and Posterior decoding are notably distant from the true values. For the case of the longest fragment, classical decoding methods infer erroneously a much longer fragment by joining three true fragments located in a different genomic region, whereas sampling from the posterior recovered a distribution corresponding to three potential longest fragments including the true longest fragment (Material S1).

The accuracy in estimating the summary distribution with this sampling based method is limited by the number of simulations performed. Instead, the analytical distribution of summary statistics can be computed using the Finite Markov Chain Imbedding (FMCI) (Aston and Martin 2007). This method consists of designing a first order Markov chain to compute the pattern of interest in which its transition probabilities are defined by the inhomogeneous transition probabilities introduced above (Materials and methods, Material S2, (Bæk et al. 2025). The flexibility of this method to define any kind of Markov chain, enables us to build a framework to compute the distribution for the three summary statistics (Fig. 3).

Depending on the specifics of each statistic, the FMCI varies in complexity, thus affecting the running time and memory efficiency of the algorithm (Material S2). While computing the number of archaic fragments takes ∼20 min for the entire 1Gb-simulated sequence (Material S2), it is infeasible to compute the distribution of the total amount of archaic sequence. Instead, we decided to divide the sequence into 100 10Mb chunks and compute the summary for each chunk in parallel. This way, we both reduce the maximum value to be computed (matrix size, *l*) and the sequence analyzed (number of iterations, *t*) ending up with a <2 min running time for each genomic chunk, which are able to run in parallel. To merge the results from each chunk together, we convolve the obtained distributions into a single one. To compute the maximum fragment length, we designed a different strategy. This is because the matrix computed scales quartically with the maximum length to be calculated ($l^{4}$, Material S2). To be able to run this algorithm, we compute sections of the distribution independently. We manage to run each job in <4 h. Finally, the resulting partial distributions are convolved together.

**Figure 4:**
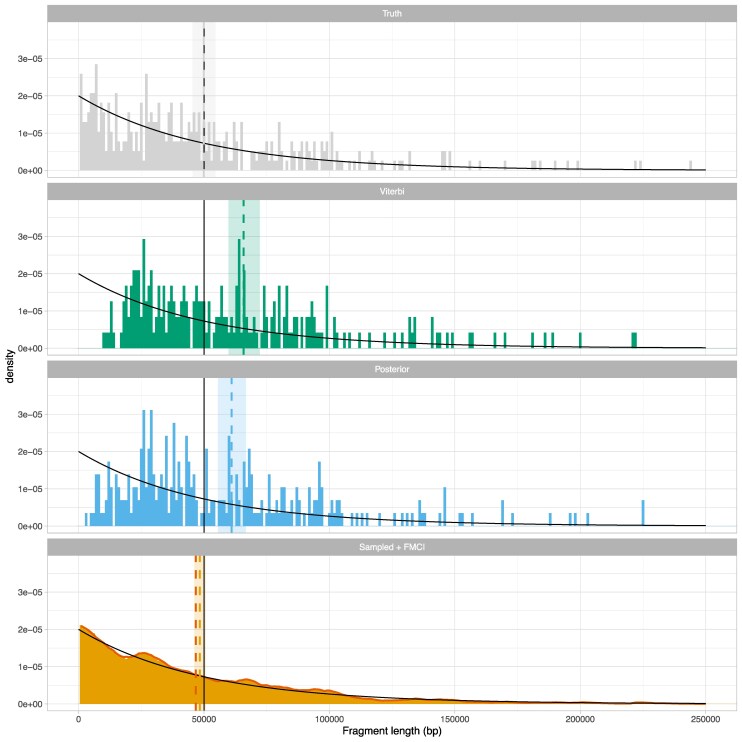
The length distribution of archaic fragments based on realistic parameter simulations for the true fragments, the Viterbi and Posterior decoded along with 1,000 paths sampled from the conditional distribution of hidden states. The FMCI distribution is plotted as a line on top of the histogram for the fragment lengths of the sampled paths. Vertical dotted lines show the mean of each distribution. The 95% CI of the mean are shown as shaded regions around the mean. For the true, Viterbi and Posterior means, the intervals are computed with bootstrap resampling (100,000 bootstraps). For the sampled paths' distribution, the mean fragment length is computed for each sampled hidden state sequence and the interval is then calculated as the 2.5 and 97.5 percentile values over all means. Note that the FMCI estimate does not have an interval. The decaying solid lines denote the theoretical length distribution of archaic fragments computed from a geometric distribution with parameter $\Gamma_{AH}\;=\;0.02$ and the vertical solid lines its mean. The plot is limited to 250,000 bp on the *x*-axis.

Both sampling from the posterior and FMCI methods can also recover specific features, such as the length distribution of archaic fragments (Fig. 4, (Bæk et al. 2025). In this case, both Posterior and Viterbi decodings also yield a distribution, but they are known to be biased since many small fragments are often missed. To compute the fragment length distribution with FMCI, we computed sections of the distribution independently due to the time complexity of the Markov chain (Material S2). We also reused the same 1,000 samples from the posterior to calculate the distribution of interest. Figure 4 compares the true fragment length distribution with the ones estimated with the various methods. As expected, classical decoding methods in general struggle to recover short fragments, with Viterbi being exceptionally worse at this. As a result, the mean fragment length is not captured by the 95% CI estimated and is instead overestimated (Viterbi mean 95% CI 59,790.79 to 72,230.13 bp, Posterior mean 55,574.31 to 66,726.64 bp, true value ∼50,000 bp). In contrast, both the sampling method and FMCI more accurately reconstruct the true distribution with an unbiased mean (sampling mean fragment length 95% CI 46,133.38 to 50,643.36 bp)

### Evaluating hmmix performance to recover summary statistics under realistic demographic parameters and scenarios

We sought to assess the robustness of hmmix to violations of the modeling assumptions that are likely to occur in empirical datasets (Coll Macià and Skov 2025). These issues are particularly relevant for human data, where demographic histories are complex and recombination rates vary across the genome, for example. We therefore carried out a suite of HMM-based and msprime (Baumdicker et al. 2021) simulations to test how well hmmix recovers summary statistics under a more realistic framework, departing from the idealized HMM assumptions (Material S3).

When emission rates are sufficiently differentiated, as expected for human–archaic introgression (ie $\lambda_{A}$ ≈ 0.3 and $\lambda_{H}$ ≈ 0.03), parameter training typically estimates $\lambda_{A}$ within 2% of its true value, and posterior sampling accurately recovers summary statistics, whereas single-path decoders remain biased (Figures S3 and S6). hmmix is sensitive to strong overdispersion in the Human state: when coalescent times vary substantially (variance/mean ≥1.125), Human-fragment emissions can overlap with expected Archaic emission distribution, affecting summary-statistic estimation (Figure S4). In practice, however, empirical data show much lower overdispersion (variance/mean <1.03, Table S4), and our simulations indicate that exceeding the problematic threshold would require unrealistically large ancestral modern human population sizes (Table S4). In addition, hmmix yields comparable estimates from haploid and diploid data, provided the appropriate decoding option is used and data are processed consistently (Figure S14).

**Figure 5:**
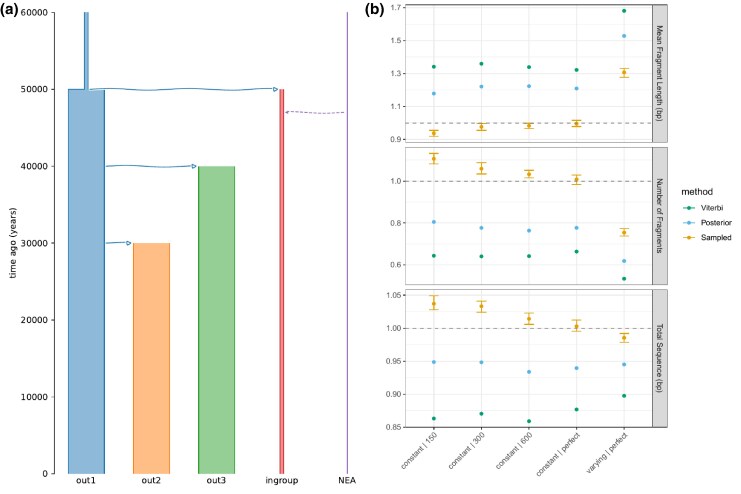
Estimation of archaic introgression summary statistics under realistic conditions. a) Demographic model for msprime simulations (scenario f) in Material S3). b) Summary-statistic estimates across simulation settings that vary recombination rate (constant vs varying) and common variation filtering efficiency (perfect filtering or conditioned to outgroup sample sizes of 50, 100, or 200 individuals sampled per population). Methods are color-coded. The *y*-axis shows the logarithm of the estimated summary statistic divided by the true value. Horizontal gray dashed lines at 1 indicates perfect agreement. For the Samped summaries, each statistic is computed for each of the 100 sampled hidden state sequences and the dot represents the mean over all the sampled paths and the 95% CI are showing the 2.5 and 97.5 percentile values.

The most challenging misspecifications are those that induce departures from geometric fragment lengths (Figure S6). While hmmix is robust to multiple admixture pulses (Figures S8 and S13), the method has limited power to detect the very short fragments produced under realistic recombination maps (Fig. 5, Figures S7 and S13). Many of these extremely short archaic fragments contain few or no SNPs and are therefore intrinsically undetectable (Material S3). Accurate inference also requires effective outgroup-based filtering of common variants: removing >95% of shared variants yields well-estimated summary statistics (Figure S9), and in demographic settings that match empirical Human state emission levels (Figure S10, Table S5), outgroup sample sizes in the range available for real datasets (eg ∼500 individuals) are sufficient for posterior sampling to recover mean fragment length, total archaic sequence, and fragment counts—improving upon Viterbi and Posterior decoding estimates (Figures S11 and S12).

To ensure these conclusions extend beyond stylized simulations, we also evaluated hmmix under a published, human-like demographic model that includes multiple introgression pulses, population structure, and migration among groups (Jacobs et al. 2019). Under this setting, posterior sampling again recovered the target summary statistics with outgroup sample sizes on the order of 600, with the expected loss of performance under variable recombination—most noticeably in mean fragment length, consistent with the reduced detectability of very short fragments (Figure S13).

### Hybrid decoding

While our conditional probabilities approaches for estimating summary statistics offers an avenue for admixture parameter inferences, the precise decoding of archaic fragments remains essential for more localized analyses. For example, accurately determining whether a specific gene in modern human genomes has been inherited from an archaic lineage requires a reliable sequence decoding.

Previous work (Lember and Koloydenko 2014; Kuljus and Lember 2023) proposed a hybrid decoding method that combines the strengths of both Viterbi and Posterior decoding (Materials and methods). We reformulate the original method by calculating the weighted geometric mean between Viterbi and Posterior decoding, and parametrizing the weights of each term with α∈[0,1] (Materials and methods, Bæk et al. 2025). Specifically, when *α* = 1 the hybrid decoding outputs the Viterbi solution and when *α* = 0, the Posterior decoding solution. Hybrid decodings are obtained with intermediate *α* values (Materials and methods).

**Figure 6:**
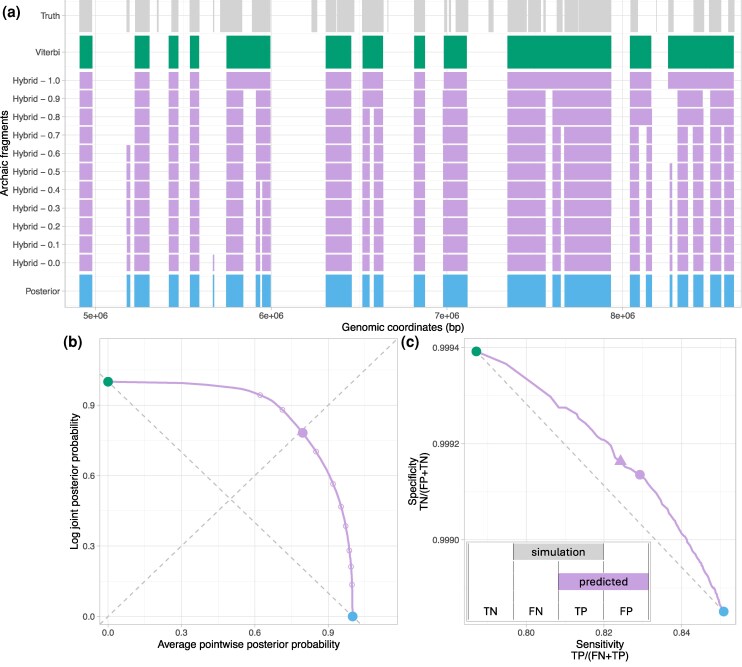
Hybrid decoding. a) Visualization of decoded paths using the hybrid method. The genomic region is the same as in Fig. 2, based on visualization parameters. The values of *α* ranges between 0 (Posterior decoding) and 1 (Viterbi) in steps of 0.1. b) Artemis plot on realistic simulation of hybrid decoding paths in a grid search for *α* (min value = 0, max value = 1, step size = 0.01), which determine the bow-shaped path. Viterbi and Posterior decoding values are shown as dots in the upper-left and lower-right extremes, respectively. *α* values ranging from 0.1 to 0.9 in 0.1 steps are shown as empty dots. The optimal *α* is shown as a solid dot. c) same as b) but for sensitivity (TN/(FP + TN)) and specificity (TP/(FN + TP)) values based on a simulation of 10^6^ 1 kb windows of the trained realistic parameters. Optimal *α* found with the Artemis analysis is shown as a dot. Optimal *α* found based on sensitivity and specificity values is shown as a triangle. The insert shows a diagram of two overlapping archaic fragments (two 1 kb windows each) to define the different terms to compute sensitivity and specificity. TN, true negatives; FP, false positives; TP, true positives; FN, false negatives.

We run hybrid decoding with hmmix's implementation (Materials and methods) on the visualization parameters’ simulated data with values of *α* ranging from 0 to 1 in increments of 0.1 units (Fig. 6). In Fig. 6, there are some examples in which Posterior decoding infers multiple fragments that are also inferred by the hybrid method with small *α* values. Those sequences transition to more unified fragments in hybrid decodings with increasing *α* values. Eventually, the hybrid estimates converge to the Viterbi solution which often joins multiple true fragments into a single path, emphasizing the importance of choosing *α* adequately.

To learn *α*, we focus on the strengths of Viterbi and Posterior decoding: the log joint posterior probability and the average pointwise posterior probability, respectively (Materials and methods, Material S4). To check how the two statistics behave, we perform a grid search of the parameter space of *α* in 0.01 increments and obtained the corresponding hybrid decoding paths (Fig. 6b, Materials and methods). The obtained results are not uniformly distributed between the extremes defined by Posterior decoding and Viterbi statistics (Fig. 6b), with a majority of the hybrid decodings resembling the Posterior decoding solution. Here, we define *α* to be optimal for hybrid decoding when its solution is balanced considering the two statistics, maximizing both simultaneously. In other words, the optimal *α* corresponds to the hybrid solution that lies closest to the 45° line between the maximum and minimum of both axes. This analysis—which we name Artemis due to the plot's bow-shaped curvature and arrow-like 45° line—identifies an optimal *α* = 0.75 (Materials and methods). We recommend running the Artemis analysis to optimize *α* in every case since the optimal point changes depending on the HMM parameters and structure (Bæk et al. 2025). Artemis analysis is conveniently implemented in hmmix (Materials and methods).

Next, we study the advantages of the hybrid decoding method in terms of specificity and sensitivity. For that, we implemented a function in hmmix that simulates a new dataset based on a set of HMM parameters, and obtains the confusion matrix by comparing to the true sequence of hidden states for all the hybrid decodings in a grid search of *α*, similar to the Artemis analysis (Materials and methods). Figure 6c shows the results of sensitivity and specificity on a simulation of 10^7^ 1 kb windows. Posterior decoding is the most sensitive but least specific method, while Viterbi is the most specific but least sensitive. The paths obtained with increasing values of *α* range between the Posterior decoding and the Viterbi results. However, those paths are not in a straight diagonal line, which would correspond to an equal tradeoff in terms of specificity and sensitivity between the two extremes. Instead, the path is bowed-shaped, denoting a better performance for hybrid decodings in general. More specifically, we can see that the results corresponding to the optimal *α* found with Artemis analysis (*α* = 0.76) is more balanced between the two extremes. Nonetheless, the *α* value that maximize both sensitivity and specificity is higher (*α* = 0.81) and thus results in a hybrid decoding that is more Viterbi-like. This tendency is confirmed in ten replicates of the same analysis (Material S5).

Overall, the hybrid implementation improves the hmmix archaic fragment inference by considering neighboring signals in an intermediate scale between the local and global extreme choices. This hybrid method reduces the relative proportion of false archaic fragments detected by Posterior decoding (sensitivity) while detecting more true archaic sequence than Viterbi (specificity), making the new decoding results more reliable. We suggest choosing the *α* that leads to the most balanced hybrid decoding by performing the Artemis analysis introduced here. Nonetheless, the hybrid method is flexible and *α* can be chosen depending on the user requirements by giving more weight to either log joint posterior probability or average pointwise posterior probability of the hidden path (Bæk et al. 2025)

### Sampling from the posterior and hybrid decoding methods applied to real data

Previous studies have reported that East Asians possess, on average, ∼20% more Neanderthal ancestry than Europeans (Sankararaman et al. 2014; Vernot and Akey 2014; Coll Macià et al. 2021). Additionally, differences in the length distribution of archaic fragments among Eurasian populations are linked to variations in ancestral generation times across these groups, as shorter archaic fragments imply more rounds of recombination—thus, more generations—since the admixture event (Coll Macià et al. 2021).

**Figure 7:**
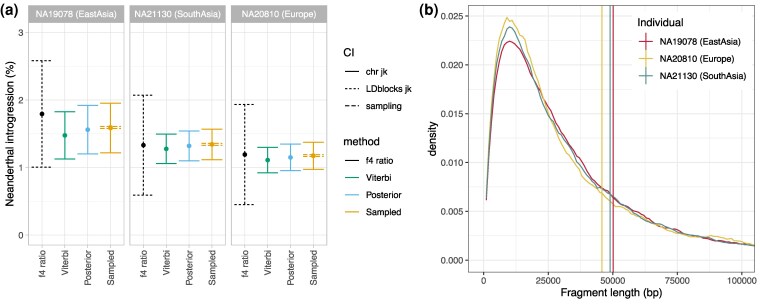
Archaic fragment summary statistics in three individuals a) Amount of Neanderthal ancestry in the phased genome using the f4 ratio statistic and three different hmmix decoding schemes. Error bars represent 95% CI of the mean for each estimate. For f4 ratio, the variance is estimated with jackknife with 0.050 cM block size (714 blocks). For HMM-based methods the variance is estimated with jackknife by chromosome. For the samples from the posterior, the 95% CI based on sampling corresponds to the 0.025 and 0.975 percentile values of the distribution sampled. b) Length distribution of introgressed archaic fragments for three individuals truncated at 100 kb. For each individual we performed 1,000 inhomogeneous samples. The vertical lines represent the mean fragment length for all 1,000 samples. Only fragments with at least one observation are used.

We sought to quantify more precisely these observations on Neanderthal ancestry proportions and fragment lengths using the sampling approach in hmmix applied to real data (Materials and methods). For these analyses, we selected one representative individual from each of three major Eurasian populations within the 1000 Genomes Project dataset: NA19078 (Japanese in Tokyo, Japan [JPT] with predominantly East Asian ancestry), NA20810 (Toscani in Italia [TSI] with predominantly European ancestry) and NA21130 (Gujarati Indians in Houston, Texas, USA [GIH] with predominantly South Asian ancestry). After archaic fragments are decoded for each individual, we annotate with hmmix the number of shared variants with sequenced archaic genomes per fragment. To focus on Neanderthal content, we select those fragments that share a majority of variants with Neanderthals and not the Denisovan genome (Materials and methods). Often, Neanderthal ancestry proportions are quantified using the direct f4 ratio method (Petr et al. 2019; Bergström et al. 2020) (Materials and methods). Our hmmix-based estimates of Neanderthal introgression for all three decoding methods are consistent with the broad confidence intervals reported by f4 ratio. Among hmmix decodings, sampling from the posterior consistently produces higher estimates which are closest to the f4 ratio mean (Fig. 7a, Materials and methods). This is consistent with our simulation analyses above pointing toward Viterbi and Posterior decoding underestimating the summary (Fig. 7a). Notably, sampling from the posterior recapitulates the higher Neanderthal ancestry proportion previously observed in East Asians (1.59%, 95% CI: 1.58% to 1.61%) compared to Europeans (1.18%, 95% CI: 1.16% to 1.19%). This difference would not be statistically detectable using the f4 ratio, as its variance estimates are based on fewer data and are derived using the conservative block-jackknife method. A similar issue arises when variance is estimated by jackknifing across chromosomes for decoding-based methods (Fig. 7a).

It is important to note that confidence intervals obtained from the posterior sampling approach are calibrated under the assumptions of the HMM. Consequently, they may be overly narrow if model parameters are misspecified or if underlying assumptions are violated (Material S3).

We also analyzed the samples from the posterior to estimate archaic fragment length distributions among the selected individuals (Fig. 7b). While the mean fragments length is likely overestimated due to recombination not being uniform along the genome (Material S3, Figure S7) we can still investigate the difference between individuals assuming the overall recombination map is similar across populations. Average fragment lengths are 45.81 kb (95% CI: 45.78 to 45.84 kb) for the European individual, 48.95 kb (95% CI: 48.92 to 48.98 kb) for the South Asian individual, and 50.14 kb (95% CI: 50.11 to 50.17 kb) for the East Asian individual. These fragment lengths are shorter than those previously reported by Coll Macià et al., likely due to our analysis using phased rather than diploid data and the greater sensitivity of sampling from the posterior for detecting shorter fragments compared to the Posterior decoding used in that study (Coll Macià et al. 2021). Nevertheless, assuming reasonable parameters for recombination rates and admixture timing, our results remain consistent with previously inferred generation time differences of approximately 1 to 3 years among Eurasian populations since their admixture event with Neanderthals (Materials and methods, Table 3).

**Table 3:** Generation time difference ($\Delta_{GT}$) estimation between an European and an East Asian individual assuming different admixture times (*a*) and recombination rates (*r*).

*a* (x10^4^ years)	*r* (x10^−8^ bp × generation)	${\underline{L}}_{EUR}$ (x10^3^ bp)	${\underline{L}}_{EAS}$ (x10^3^ bp)	$\Delta_{GT}$ (years)
6.00	0.80	45.81	50.14	2.08
4.00	0.80	45.81	50.14	1.39
6.00	1.25	45.81	50.14	3.25
4.00	1.25	45.81	50.14	2.17

Thus, a difference of 1 to 3 years of generation time since the admixture event.

**Figure 8:**
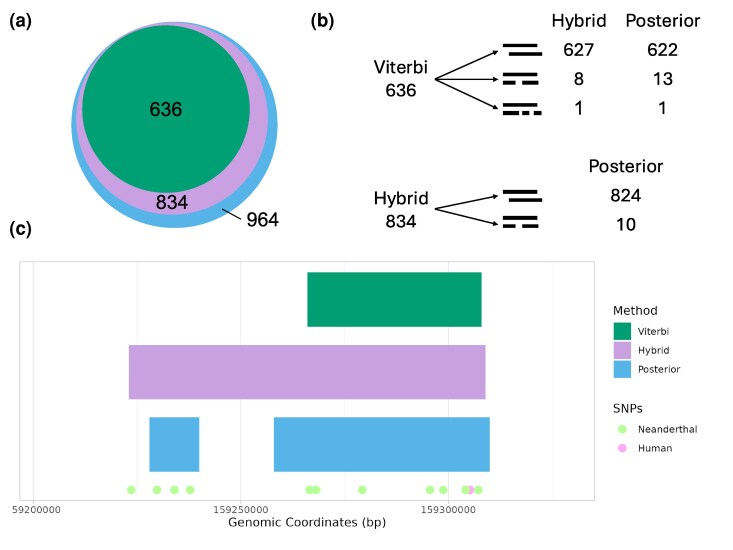
Hybrid decoding for individual NA19078, haplotype 1, chromosome 1. a) Venn diagram of the archaic fragments found by each decoding method. b) Number of fragments from Viterbi (above) and hybrid (below) that are found by a single (one to one fragment) or multiple fragments (one to two or one to three fragments) by the methods in the columns (hybrid and/or Posterior decoding). Fragments are considered to be found by another method fragment if they overlap by at least 1 bp. c) Local archaic fragment calling for a region in which there are differences in calling among methods. Decoded fragments for each method are shown as horizontal bars. Observed genetic derived variants are shown as circles. Variants shared with any of the high coverage Neanderthal genomes are colored accordingly (no derived variant is shared with the Denisovan genome). Variants not found in any archaic genome are colored as Human.

Next, we apply hybrid decoding with their corresponding optimal *α* value to the three individuals (NA19078 *α* = 0.81; NA20810 *α* = 0.82; NA21130 *α* = 0.83). We then compare the identified archaic fragments with those obtained previously using Viterbi and Posterior decoding. We observe a clear nested relationship among the three methods: Posterior decoding consistently identifies the most fragments, encompassing all fragments found by hybrid and Viterbi decoding, while hybrid decoding similarly includes all fragments detected by Viterbi in all haplotypes of the three individuals (Fig. 8a, Material S6). A small proportion (<5%) of fragments called by Viterbi decoding are subdivided into two or three smaller fragments by hybrid and/or Posterior decoding, indicating the more local resolution of these methods. Likewise, hybrid fragments were occasionally (<1.5%) further subdivided by Posterior decoding (Fig. 8b, Material S6). Figure 8c provides an example of such cases in which the boundaries of archaic fragments identified by the three decoding methods differ significantly. The example involves a 126 kb genomic region on chromosome 1 of individual NA19078. This region contains two distinct clusters of derived variants shared with Neanderthals, upstream and downstream of 159.25 Mb. Viterbi decoding identifies only the second cluster as archaic, whereas Posterior decoding identifies two separate archaic fragments corresponding to each cluster individually. In contrast, hybrid decoding infers a single archaic fragment spanning both clusters. A priori, none of the three methods is superior at calling edges of archaic fragments on average (Material S7). In this particular case, given the high linkage disequilibrium (R^2^ ∼0.8, Materials and methods, Material S8) observed among the Neanderthal-shared variants in the flanking regions of these two clusters, the scenario suggested by hybrid decoding appears more plausible. This scenario suggests that both clusters represent parts of a single Neanderthal haplotype in contrast to Viterbi decoding which only identifies a fragment localized in the second cluster. Moreover, the Posterior decoding scenario with two fragments implies multiple recombination events within this limited genomic region, making it a less parsimonious explanation due to the high LD observed.

## Discussion

In this manuscript, we introduce two novel enhancements to hmmix which improve the detection of archaic fragments and estimation of summary statistics relevant in the study of the admixture events between modern and archaic humans. An updated version of hmmix incorporating these enhancements—for both haploid and diploid data—is available on https://github.com/LauritsSkov/Introgression-detection.

The first enhancement focuses on improving the estimation of summary statistics with an analytical and empirical technique based on conditioning the transition probabilities between hidden states on the observed data. FMCI provides exact analytical distributions for summary statistics but becomes computationally intensive at genome-wide scales. Calculating archaic fragment length distribution or the longest fragment distribution can become infeasible when analyzing datasets as in this manuscript, containing millions of observations. To overcome these computational challenges, we have divided and parallelized computations. However, future research should investigate more efficient strategies, such as adaptive matrix sizing or sparse matrix approaches. As for now, the dimensions of FMCI matrices must be pre-set based on parameters typically approximated with sampling from the posterior—an approach that introduces some circularity, given that these empirical summaries already serve this purpose.

In general, posterior sampling is computationally more efficient and the same hidden state samples simultaneously provide all summary statistics. We show how this approach significantly improves precision, particularly in estimating the proportion of Neanderthal (archaic) ancestry with individual genomes. Moreover, posterior sampling naturally provides a measure of uncertainty, allowing variance around summary statistics to be quantified. However, the resulting confidence intervals are only accurate if the model is correctly specified and its parameters are well estimated. As illustrated in Figure S3, even when parameter estimates are suboptimal, the posterior sampling approach still yields overly confident estimates. Therefore, it is important to assess the adequacy of parameter estimates and decoded sequences, for example by inspecting posterior probabilities of decoded sequences or validating results with simulated data, to ensure robust interpretation of the inferred statistics.

Sampling from the posterior captures shorter and less probable fragments among all samples, which are never represented in Viterbi nor Posterior decoding, due to the lack of probabilistic support. Sampling from the posterior substantially reduces bias, specifically in fragment length summaries estimation. With the correct fragment length distribution it will enable more accurate and direct inference of admixture times with archaic populations in future studies. Finally, estimates derived from these approaches demonstrate robustness to violations of classical HMM assumptions, such as non-geometric distributions of fragment lengths, which can occur due to variable recombination rates or recent admixture events (Liang and Nielsen 2014).

The second enhancement, hybrid decoding, effectively combines the advantages of Posterior decoding and Viterbi. By adjusting the *α* parameter, researchers can smoothly transition between local (Posterior decoding) and global (Viterbi) decoding strategies, enabling optimal customization to specific analytical needs. The Artemis plot reveals that hybrid decoding successfully combines global decoding with local probabilistic precision, significantly reducing both false-positive and false-negative fragment identifications. This flexibility is particularly advantageous in targeted genomic analyses, facilitating informed assessments of archaic ancestry in particular haplotypes through decoding strategies positioned between the traditional Posterior decoding and Viterbi solutions.

Although specifically developed for hmmix, these methods can be adapted to any HMM framework. We anticipate these methodological enhancements will become widely adopted analytical tools, benefiting various HMM-based genomic studies and other fields.

## Materials and methods

### Simulations

We introduce two distinct scenarios, each characterized by specific assumptions that constrain the parameters of the two-state Poisson HMM in hmmix (Fig. 1): emission probabilities (*Φ*), starting probabilities (*π*), and transition probabilities (*Γ*).


$$\Phi \;=\;(\Phi_{H},\;\Phi_{A})\;\;\;\;\;\;\;\;\pi \;=\;(\pi_{H},\;\pi_{A})\;\;\;\;\;\;\;\;\Gamma \;=\;[\left(\Gamma_{HH},\;\Gamma_{HA}),\;(\Gamma_{AH},\;\Gamma_{AA}\right)]$$

where *H* denotes the human state and *A* denotes the archaic state. Note that *Φ* is determined by the Poisson rates $\lambda \;=\;(\lambda_{H},\;\lambda_{A})$ in the case of hmmix. From these HMMs data is simulated and analyzed.

#### Realistic parameters

The realistic set is calculated such that the mean archaic fragment length is 50 kb and the archaic proportion genome wide is 2%.

The first assumption defines the transition probability between archaic states ($\Gamma_{AA}$), since the archaic fragments are geometrically distributed with rate $1-\Gamma_{AA}\;$. Thus, the mean of that distribution is


$$\frac{1}{1-\Gamma_{AA}}\;=\;50\Rightarrow \;\;\;\;\Gamma_{AA}\;=0.98\;.$$

Additionally, we have that


$$\Gamma_{AH}\;=1-\Gamma_{AA}=0.02.$$

The second assumption defines the starting parameters $\pi_{A}\;=\;0.02$ and $\pi_{H}\;=\;0.98$. Since the starting probabilities (*π*) are the stationary distribution of the Markov chain, it holds that


π=πΓ.

Together with $\Gamma_{AA}$, we obtain the remaining parameters with


$$\pi_{A}\;=\pi_{H}\Gamma_{HA}+\pi_{A}\Gamma_{AA}\;\Rightarrow \;\;\;\;\Gamma_{HA}\;=\frac{\pi_{A}-\pi_{A}\Gamma_{AA}}{\pi_{H}}\;=\;0.000408,$$

and


$$\Gamma_{HH}\;=1-\Gamma_{HA}\;=\;0.9995918.$$

Finally, the emission parameters—which depend on the divergence time between modern humans and archaics (archaic state emissions) and the divergence time between African and non-African genomes (human state emissions)—are obtained from literature (Skov et al. 2020) and set to $\lambda_{A}\;=\;0.3\;$ and $\lambda_{H}\;=\;0.03$.

These parameters are presented in Table 1 and saved in the file realistic_param.json.

#### Visualization parameters

The second set of parameters increases the overall amount of archaic sequence (1/3) and shortens the length of human fragments (mean length = 100 kb). We also make the emission probabilities more similar between the two states so that decoding methods have less power to detect archaic fragments.

This way, we obtain more archaic fragments overall and more differences among decoding methods for visualization purposes. These parameters are presented in Table 2 and saved in the file visualization_param.json.

Simulated data of a single chromosome of 1 × 10^6^ 1 kb-windows (genomic sequence of 1 Gb) are generated with the hmmix command hmmix make_test_data for both realistic and visualization parameters.

Parameters are estimated with the implemented Baum-Welch algorithm in hmmix by running the command hmmix train (Tables 1 and 2). We use the estimated parameters for all the analyses in the manuscript.

The 95% CI for parameter estimation is obtained by parametric bootstrapping. For that, we simulate data from the parameter estimates (Table 1) and re-estimating parameters using the Baum-Welch algorithm 100 times with hmmix (hmmix make_test_data and hmmix train commands). The interval is obtained for each parameter as the 2.5% and 97.5% percentile of the 100 parameter values estimated. The 95% CIs are very narrow, with little variation and capture the true value (considering up to four decimal digits). This reflects that there is little uncertainty in the parameter estimation in the simulated data by hmmix for the amount of data simulated and assuming that these parameters are correct.

The exact commands and relevant files are provided in the simulated data and the real data directories on the GitHub repository (https://github.com/MoiColl/HMMenhancements).

### Sampling from the posterior

As shown in (Bæk et al. 2025), the hidden state sequence *y* conditional on the observed sequence *x* is an inhomogeneous Markov chain with transition probabilities


$$P(y_{t}\;|\;y_{t-1},\;y_{t-2},\;\ldots ,\;y_{1},\;x)=P(y_{t}\;|\;y_{t-1},\;x_{t},\;\ldots ,\;x_{n})=\frac{\beta_{t}(y_{t})\Gamma_{y_{t-1},y_{t}}\Phi_{y_{t}}(x_{t})}{\beta_{t-1}(y_{t-1})}$$

for t=1,…,n where $\beta_{t}(y_{t})=P(x_{t+1},\;\ldots ,\;x_{n}\;|\;y_{t})$ is the matrix of backward probabilities from the Posterior decoding framework, *Γ* is the matrix of transition probabilities and *Φ* is the emission probabilities. Note that *n* is the last index in the sequence in a zero based index and not the total number of windows (*m*), thus, first index in the sequence *t* = 0 and n=m−1). In the case of hmmix, the emission probabilities *Φ* correspond to the Poisson rates *λ*, such that $\Phi_{A}(x_{t})\;=Poisson(x_{t};\;\lambda_{A})$ and $\Phi_{H}(x_{t})\;=Poisson(x_{t};\;\lambda_{H})$, which are the probabilities of observing *x* SNPs in position *t* under a Poisson distribution in the archaic state *A* and the human state *H*, respectively.

The initial distribution for t=0 of the inhomogeneous Markov chain is


$$P(y_{0}\;|\;x)\;=\;\frac{P(y_{0},\;x)}{P(x)}\;=\;\frac{\beta_{0}(y_{0})\pi_{y_{0}}\Phi_{y_{0}}(x_{0})}{P(x).}$$

See (Bæk et al. 2025) for the proof of the theorem above. Note that here the first index is 0 while in (Bæk et al. 2025) the first index is 1. This is because the code provided in this manuscript is written in python.

The probabilities of staying in the human state conditional on the data $P(\;y_{t}=\;H\;|\;y_{t-1}=\;H,\;x_{t},\;\ldots ,x_{n}\;)$ are denoted throughout the paper as $a_{t}$ and the probability of leaving the human state $P(\;y_{t}=\;A\;|\;y_{t-1}=\;H,\;x_{t},\;\ldots ,x_{n}\;)$ as $1-a_{t}$. Similarly, the probabilities of staying in the archaic state conditional on the data $P(\;y_{t}=A\;|\;y_{t-1}=\;A,\;x_{t},\;\ldots ,x_{n}\;)$ are denoted as $b_{t}$ and the probability of leaving the archaic state $P(\;y_{t}=\;H\;|\;y_{t-1}=\;A,\;x_{t},\;\ldots ,x_{n}\;)$ as $1-b_{t}$. Similarly denoted, the initial values $P(y_{0}=\;H\;|\;x\;)$ are denoted $a_{0}$ and $P(y_{0}=\;A\;|\;x\;)$ as $b_{0}$.

Once the HMM parameters have been estimated and given the observed sequence, these inhomogeneous transition probabilities can be used to sample hidden state sequences.

The calculation of these inhomogeneous transition probabilities, as well as sampling hidden state sequences, are implemented in hmmix with the command hmmix inhomogeneous. In this study, 1,000 sequences are sampled for both realistic and visualization simulated scenarios and for each real data individual.

The exact commands are provided in the simulated data and the real data directories on the GitHub repository (https://github.com/MoiColl/HMMenhancements).

### Finite Markov chain imbedding

The finite Markov chain imbedding (FMCI) framework consists on building a Markov chain for a specific pattern of interest at every point in the sequence based on the inhomogeneous transition probabilities $a_{t}$ and $b_{t}$. Once the chain is encoded in a matrix $\Delta (a_{t},b_{t})$ for every point in the sequence (*t*), we can obtain the probability distribution of the summary of interest with


$$\eta \overset{n}{\underset{t=1}{\prod }}\;\Delta (a_{t},b_{t}).$$

Here *η* represents the starting vector consisting of $a_{0}$ and $b_{0}$. The resulting vector will encode the probability distribution of the summary of interest for the sequence analyzed.

The number of archaic fragments as an example is explained in detail in Material S2. The FMCI constructed to compute the number of archaic fragments (number of jumps), the total archaic sequence (number of states), the maximum archaic fragment length (longest run) and the archaic fragment length distribution (run length distribution) are detailed in (Bæk et al. 2025). The running time for all algorithms as well as strategies to parallelize and decrease running time are explained in Material S2.

The FMCI scripts are provided in the FMCI directory on the GitHub repository (https://github.com/MoiColl/HMMenhancements).

### Hybrid decoding

Viterbi decoding infers the global hidden state sequence *s* that maximizes P(y=s|x), where *x* is the observed sequence and *y* the hidden state sequence


$$s\;=\;argmax_{u}P(y\;=\;u\;|\;x\;)$$

Posterior decoding infers the local hidden state sequence $s_{t}$ that maximizes $P(y_{t}\;=\;s_{t}\;|\;x\;)$ for every position *t* in the sequence


$$s_{t}\;=\;argmax_{u_{t}}P(y_{t}\;=\;u_{t}\;|\;x\;)$$

The hybrid decoding method mixes Posterior decoding and Viterbi methods by combining the logarithm function both in the same equation as


$$s\;=argmax_{u}\left\{\alpha \;log(P(y\;=\;u\;|\;x\;))+(1-\alpha )\overset{n}{\underset{t=0}{\sum }}\;log(P(y_{t}\;=\;u_{t}\;|\;x\;))\right\}\;$$

where α∈[0,1] weights the two terms in the equation resulting in the same solution as Posterior decoding when α=0, the same solution as Viterbi decoding when α=1 and intermediate values correspond to hybrid solutions.

The *α* parameter can be chosen using the Artemis analysis based on log joint posterior probability and average pointwise posterior probability (Material S4) as described in the main text of this paper and (Bæk et al. 2025). The hybrid path for a given *α* can be calculated efficiently using a recursive algorithm similar to Viterbi (Kuljus and Lember 2023; Bæk et al. 2025). The full overview of the method and its derivation can be found in (Bæk et al. 2025).

The hybrid decoding method is implemented in hmmix using the command hmmix decode -hybrid. The pipeline to find the optimal *α* using Artemis analysis is also implemented in hmmix with the command hmmix artemis. To obtain the confusion matrix of a simulated dataset, hmmix artemis_sim conveniently simulates, decodes and compares the true sequence with the decoded sequence in the same function. The exact commands are provided in the simulated data's jupyter notebook and the real data's workflow.py on the GitHub repository (https://github.com/MoiColl/HMMenhancements).

**Table:** Real data sources

**1000 Genomes Project and HGDP datasets jointly called and phased bcfs**
Zan Koenig et al. Genome Research, 2024 (Koenig et al. 2024)
Phased bcf files from which three individuals were used as hmmix ingroups
gs://gcp-public-data--gnomad/resources/hgdp_1kg/phased_haplotypes_v2/
**1000 Genomes Project and HGDP polymorphic outgroup variants**
Laurits Skov. Zenodo, 2024 (doi.org/10.5281/zenodo.11212339)
Text file with the list of variants found polymorphic in a set of Sub-Saharan African individuals used as outgroup
https://zenodo.org/records/11212339/files/hg38_Outgroup_1000g_HGDP.txt
**Helper files for hmmix pipeline for hg38 assembly**
Laurits Skov. Zenodo, 2024 (doi.org/10.5281/zenodo.11212339)
Files containing information about the reference allele, the ancestral allele, the stick callability mask and the mutation rate for the hg38 coordinate system. The mutation rate was originally obtained running hmmix mutation_rate from the outgroup txt file above.
https://zenodo.org/records/11212339/files/hg38_refgenome.tar.gz https://zenodo.org/records/11212339/files/hg38_mutationrate.bed https://zenodo.org/records/11212339/files/hg38_ancestral.tar.gz
https://zenodo.org/records/11212339/files/hg38_strick_callability_mask.bed
**Archaic vcfs and bed filter files**
Laurits Skov. Zenodo, 2024 (https://zenodo.org/records/13368126)
Chagyrskaya, Vindija, Altai Neanderthals and Denisovan archaic individuals’ bcf for annotating observation variants in ingroup individuals. These variants were originally called with snpAD (Prüfer 2018) and lifted over from hg19 to hg38 with UCSC liftover tool (Perez et al. 2024).
https://zenodo.org/records/13368126/files/individuals_highcov.1.bcf https://zenodo.org/records/13368126/files/individuals_highcov.1.bcf.csi https://zenodo.org/records/13368126/files/individuals_highcov.2.bcf https://zenodo.org/records/13368126/files/individuals_highcov.2.bcf.csi https://zenodo.org/records/13368126/files/individuals_highcov.22.bcf https://zenodo.org/records/13368126/files/individuals_highcov.22.bcf.csi
**The Allen Ancient DNA Resource (AADR) v54.1 (dataverse version 7.0)**
Swapan Mallick. Sci Data, 2024 (Mallick et al. 2024)
Genetic variation dataset including the ingroup individuals used in this study chip sequenced for the 1240k array. We used this dataset to compute f4 ratio statistics.
https://dataverse.harvard.edu/dataset.xhtml?persistentId=doi:10.7910/DVN/FFIDCW&version=7.0

### Direct f4 ratio statistic

To compute the f4 ratio we used qpF4ratio (https://github.com/DReichLab/AdmixTools). We used the AADR dataset.

We run the statistic


$$\frac{\;f_{4}(Altai,\;Chimpanzee;\;X,\;Mbuti)}{\;f_{4}(Altai,\;Chimpanzee;\;Vindija,\;Mbuti),}$$

where *X* is any of the three test individuals NA19078, NA20810 and NA21130. The exact commands are given in the real data's jupyter notebook on the GitHub repository (https://github.com/MoiColl/HMMenhancements).

### Historical generation time comparison among individuals

The archaic fragment length distribution (*L*) of a given individual is exponentially distributed and the rate of decay (*λ*) corresponds to the number of generations since the admixture event (*g*) and the recombination rate per generation (*r*), thus


L∼Exp(gr)

Since the mean of an exponential distribution is the inverse of its rate, we have that the mean archaic fragment length can be calculated as


$$E[L]=\;\frac{1}{gr}$$

We can rewrite $g\;=\;\frac{a}{GT}$, where *a* is the years since the admixture event and *GT* is generation time. Thus, given E[L], *a*, and *r*, we can get *GT* with


GT=E[L]ar

The expected fragment length can be approximated with the average fragment length $\bar{L}$


$$\bar{L}\Rightarrow E[L]$$

To know the difference in generation time between population *X* and population *Y*, we can compute that with


$$\Delta_{GT}=GT_{X}-GT_{Y}\;=\;{\bar{L}}_{X}ar\;-\;{\bar{L}}_{Y}ar\;=ar({\bar{L}}_{X}-{\bar{L}}_{Y})\;$$

If we assume reasonable values for *a* and *r*, and use the mean fragment length from the European individual (NA20810) and the East Asian individual (NA19078), we get

### Linkage disequilibrium patterns

We calculated r^2^ with vcftools (v 0.1.16) (Perez et al. 2024) among East Asian individuals from the real data bcfs. The exact commands are provided in the real data's jupyter notebook on the GitHub repository (https://github.com/MoiColl/HMMenhancements).

### Code availability

The updated hmmix 1.0.0 can be installed through pipy and can be found in https://pypi.org/project/hmmix/ and https://github.com/LauritsSkov/Introgression-detection.

The scripts coded to produce data and tables, perform statistical analysis and plot figures for this manuscript are accessible on GitHub (https://github.com/MoiColl/HMMenhancements). The scripts provided in this repository are licensed under the MIT License.

## Supplementary Material

msag134_Supplementary_Data

## Data Availability

The simulated data underlying this article are available in https://github.com/MoiColl/HMMenhancements, or can be regenerated using the software in the same repository. The real datasets were derived from sources in the public domain all listed in the Methods section of this manuscript.
