# Supplementary material for: Enhancement of hidden Markov model analyses for improved inference of archaic introgression in modern humans
Source: Mol Biol Evol. 2026 Jun 3;43(6):msag134. doi: 10.1093/molbev/msag134 (PMC13274467; doi:10.1093/molbev/msag134)
Supplement: msag134_Supplementary_Data [file msag134_supplementary_data.docx]

Supplementary Material

# Enhancement of hidden Markov model analyses for improved inference of archaic introgression in modern humans

Moisès Coll Macià^1^*, Laurits Skov^2^*, Zenia Elise Damgaard Bæk^3^, Asger Hobolth^4^

1. Bioinformatics Research Centre, Aarhus University, Aarhus C, Denmark. [moicoll@birc.au.dk](mailto:moicoll@birc.au.dk) . ORCID 0000-0002-7328-3553
2. Section for Molecular Ecology and Evolution, Globe Institute, University of Copenhagen, Copenhagen, Denmark. [laurits.skov@sund.ku.dk](mailto:laurits.skov@sund.ku.dk). ORCID 0000-0001-9582-0391
3. National Centre for Register-based Research, Department of Public Health, Aarhus University, Aarhus V, Denmark.

[zenia.ncrr@au.dk](mailto:zenia.ncrr@au.dk). ORCID 0009-0008-6098-3076

1. Department of Mathematics, Aarhus University, Aarhus C, Denmark, [asger@math.au.dk](mailto:asger@math.au.dk). ORCID 0000-0003-4056-1286

* Authors contributed equally (shared first authorship)

### S1 - Longest archaic fragment

We identify the longest fragment decoded by Viterbi, Posterior decoding, and the longest fragments in the 1,000 samples from the conditional distribution of state sequences and compare them to the true longest fragment (Figure 3). We observe that classical decoding methods infer substantially longer fragments than the true one, whereas the sampling-based method produces a distribution with three distinct modes, which include the true value. This is also observed in the distribution obtained with FMCI (Figure 3, Supplementary Material S2).

Inspection of the genomic locations of these fragments reveals that the longest fragments decoded by both Viterbi and Posterior decoding methods are the same fragment found in the same genomic region, which is distant from the region containing the true simulated longest fragment (Table S1, Figure S1). In both cases, these long fragments result from the artificial joining of three adjacent true fragments.

##### Table S1. Longest fragment length statistics for the true longest fragment in simulations and the longest decoded by Viterbi and Posterior decoding.

| **Method** | **Start (bp)** | **End (bp)** | **Length (bp)** |
| --- | --- | --- | --- |
| Truth | 381,261,000 | 381,556,000 | 295,000 |
| Viterbi | 204,816,000 | 205,153,000 | 337,000 |
| Posterior | 204,814,000 | 205,154,000 | 340,000 |

##### Table S2. Longest fragment length statistics for the three groups of fragments inferred by sampling from the posterior method.

| **Start - End region (Mb)** | **Number of fragments** | **95% CI Start (bp)** | **95% CI End (bp)** | **95% CI Length (bp)** |
| --- | --- | --- | --- | --- |
| 204 - 205 | 611  (60.80 %) | 204,813,557 204,813,006 | 205,154,301 205,153,529 | 341,112.2 340,154.6 |
| 381 - 381 | 342  (34.03 %) | 381,260,132 381,259,114 | 381,560,641 381,559,394 | 301,172.8 299,616.7 |
| 431 - 432 | 52  (5.17 %) | 431,958,166 431,951,027 | 432,250,748 432,246,521 | 298,311.7 289,765.2 |

In contrast, sampling from the posterior identifies candidate fragments distributed across three distinct genomic regions (Table S2, Figure S1). The most probable fragment identified to be the longest (~61% posterior probability) is the same fragment inferred by the Viterbi and Posterior decoding methods (Figure S1a). The second fragment, located in the region of the true longest fragment, accounts for ~34% of the probability mass and is, on average, 5 kb longer than the true fragment (Figure S1b). This fragment is inferred to be the longest when the former fragment is broken into pieces. The remaining ~5% of the probability mass corresponds to a shorter fragment in a different genomic location, which is shorter than the other two inferred longest fragments (Figure S1c).


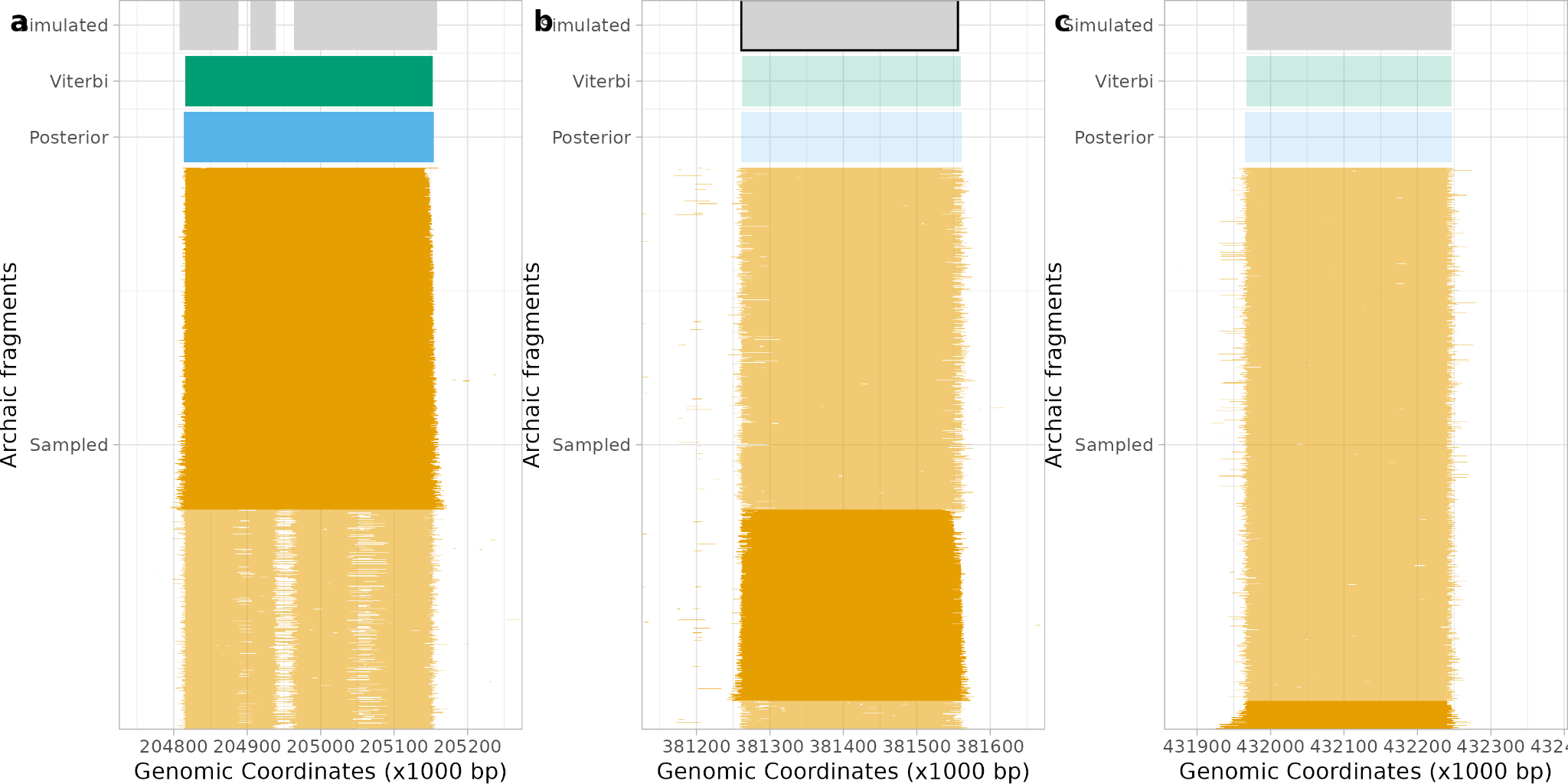


##### Figure S1. Three genomic regions where sampling from the posterior decodes the longest archaic fragments in its 1,000 samples. The archaic fragments from true (grey), Viterbi decoded (green), Posterior decoded (blue) and 1,000 samples from the conditional distribution of hidden states (orange). In solid colours are shown the decodings that contain the longest fragment in the genomic region shown in each panel. The true longest fragment in the simulated data is shown with black margins. a) Genomic region 204,750,000-205,250,000 bp. b) Genomic region 381,150,000-381,650,000 bp corresponding to the true longest fragment location. c) 431,880,000-432,380,000 bp.

In conclusion, both Viterbi and Posterior decoding incorrectly identify a much longer fragment in an incorrect location due to the merging of three adjacent true fragments. The same spurious fragment is also the most probable outcome under the sampling-based approach. Nevertheless, posterior sampling also identifies an alternative candidate corresponding to the true longest archaic fragment, although it is inferred to be ~5 kb longer on average (1.7% longer) than the true fragment. This showcases the ability of sampling from the posterior to consider multiple options, including the correct fragment for a better inference of the correct summary statistic.

### S2 - Finite Markov Chain Imbedding (FMCI)

The FMCI framework consists of building a Markov chain to compute a summary statistic of interest based on the inhomogeneous transition probabilities. Below, we explain the details of computing FMCI for the number of archaic fragments as a general illustrative example. The description of the other summary statistics can be found in [(Bæk et al. 2025)](https://app.readcube.com/library/b0a9d0bb-10df-4f1c-a9f9-fbbf1e9e85ec/all?uuid=7787746206599476&item_ids=b0a9d0bb-10df-4f1c-a9f9-fbbf1e9e85ec:e845c205-2a3f-40b9-994b-18605040de49). Here, we only discuss the running time of the algorithms corresponding to each statistic and how to speed up those by mainly parallelising computations.

#### Number of archaic fragments

Taking as an example the calculation of the number of archaic fragments in a sequence, the corresponding states of the Markov chain can be coded with two indices (Figure S2):

1. The number of archaic fragments observed (first index, starting at 0 with a maximum number of $l$ fragments)
2. Being in an archaic or human state (second index, archaic “A” or human “H”)

Considering this, there will be $2l+2$ states, plus a final absorbing state, thus a total of $2l+3$ states defining the Markov chain (Figure S2). The first two states (index 0 and 1) correspond to the probabilities of observing 0 archaic fragments. The following two states (index 2 and 3) correspond to the probabilities of observing 1 archaic fragment and so on. Finally, the absorbing state will hold the probability of observing $>l$ fragments.

The transition probabilities of the defined Markov chain can be represented with a square matrix $\Delta(a_{t},b_{t})$ in which the probabilities of transitioning from one state to another for a specific $t$ in the sequence. More specifically, the row index corresponds to the state that the transition starts from ($t-1$), and the columns correspond to the transition end ($t$, Figure S2b).


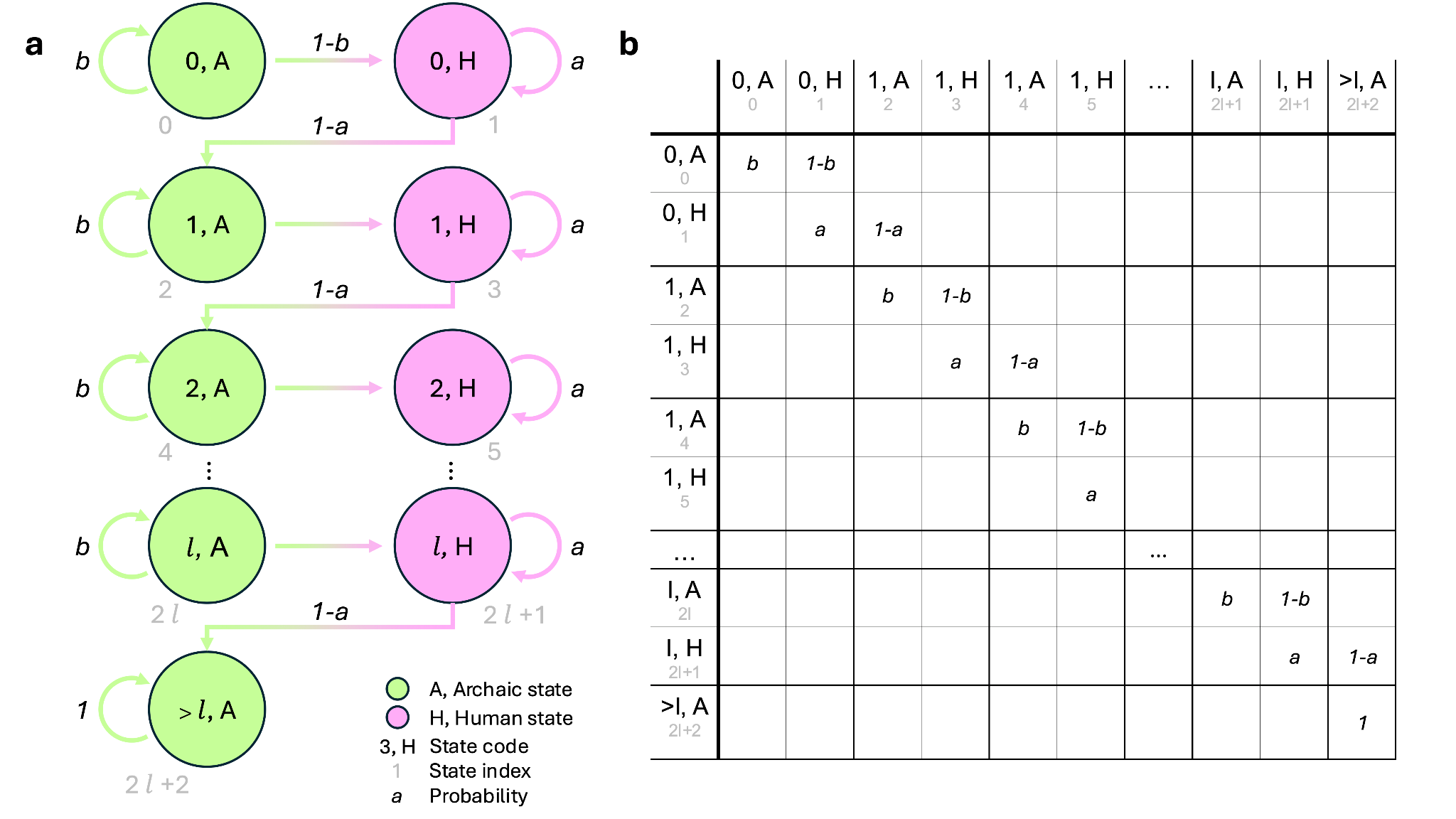


##### Figure S2. Markov chain $\Delta(a_{t},b_{t})$ representation of the FMCI method in graph form (a) and its transition probability matrix (b) to compute the number of archaic fragments in a sequence. This figure is based on figures and matrices from [(Bæk et al. 2025)](https://app.readcube.com/library/b0a9d0bb-10df-4f1c-a9f9-fbbf1e9e85ec/all?uuid=8648996664385228&item_ids=b0a9d0bb-10df-4f1c-a9f9-fbbf1e9e85ec:e845c205-2a3f-40b9-994b-18605040de49), adapted to the hmmix case. Note that in [(Bæk et al. 2025)](https://app.readcube.com/library/b0a9d0bb-10df-4f1c-a9f9-fbbf1e9e85ec/all?uuid=6054561679788282&item_ids=b0a9d0bb-10df-4f1c-a9f9-fbbf1e9e85ec:e845c205-2a3f-40b9-994b-18605040de49), states H and L denote High and Low, respectively, and do not relate to the states used here, Human (H) and Archaic (A). Also note that the state index is a 0-start-index, while the corresponding index in the [(Bæk et al. 2025)](https://app.readcube.com/library/b0a9d0bb-10df-4f1c-a9f9-fbbf1e9e85ec/all?uuid=6426772880636074&item_ids=b0a9d0bb-10df-4f1c-a9f9-fbbf1e9e85ec:e845c205-2a3f-40b9-994b-18605040de49) is 1-start-index.

Note that the transition probabilities $a$ and $b$ are time dependent, meaning that they change depending on the position $t$ in the sequence of length $m$ that is analysed.

The initialising probabilities ($t = 0$) to compute the number of archaic fragments summary statistic is $\eta= (0,a_{0}, b_{0}, 0, 0, ..., 0 )$. The first entry is defined as 0 since, according to the transition matrix, that state would correspond to starting in an archaic fragment, but counting 0 fragments. Thus, if starting in an archaic fragment, the count should directly increase to 1, which corresponds to state 2.

The posterior distribution for the number of archaic fragments for the entire sequence can be calculated from

$\eta\prod_{t=1}^{n} \Delta(a_{t},b_{t})$,

which results in a vector of length $2l+3$. Note that $n$ is the last index in the sequence of length $m$, thus $n = m-1$. The sum of the two first entries correspond to the probability of zero fragments, the sum of the next two entries is the probability of one fragment, and so on. The last entry corresponds to the probability of observing $l+1$ or more fragments.

Thus, the time complexity of this algorithm will be $O\left( ml^{2} \right)$. For simulations, $m$ is 10^6^. To reduce the computational time of the algorithm, we minimise $l$ by approximating this value with the results from sampling from the posterior, which we set to 10^4^ (Figure 3).

#### Total archaic sequence

The running time for the total archaic sequence is

$$O\left( ml^{2} \right)$$

Where $m$ is the sequence length (genome length, 10^6^ for simulations), and $l$ is the maximum archaic sequence for which the probability will be computed, which can be approximated with the samples from the posterior (~2.05x10^4^ for simulations).

Since $l$ is large, the algorithm’s running time becomes computationally infeasible to complete. Thus, we use the strategy of dividing the sequence into 100 blocks of size 10^4^ to be analysed independently. This enables us to reduce both $m$ to 10^4^ and $l$ to 10^3^, making each computation much faster. The results are then convoluted to sum all the distributions.

Note that the last probability in each block corresponds to observing $>l$ archaic sequence. When performing convolution, operations involving this last bin incorrectly attribute probability mass to specific outcomes, even though it represents an aggregate of larger, unbounded values. As a result, the probability mass in the convolved distribution may be slightly inflated. Since we choose $l$ large enough, the last bin has very little probability mass, and thus, the effect on the resulting distribution is negligible. This is also true for the archaic fragment length distribution, for which we also convolve.

#### Archaic fragment length distribution

This algorithm computes the probability distribution of observing the number of archaic fragments of extract length $k$. Once we get the probability distribution, it reports the mean number of fragments of extract length $k$. To obtain the full distribution of lengths $1,2,3, ..., K$ we must compute the mean value for every value $k$ independently. The time complexity for each computation is

$O\left( m(l{k)}^{2} \right)$,

where $m$ is the sequence length (genome length, 10^6^ for simulations), $k$ is the fragment length (up to 250), and $l$ is the maximum number of archaic fragments of exact length $k$ for which we are going to compute the probability. Similarly to the total archaic sequence, we reduce $m$ by dividing the sequence into 2 chunks to be analysed independently, and the resulting distributions are subsequently convoluted. Based on the results from sampling from the posterior distribution, we also limit $l$ to 25 for $k$ ≤ 50, to 15 for 25 < $k$ ≤ 100, to 10 for 100 < $k$ ≤ 150, to 5 for 150 < $k$, which is critical for the running time of the algorithm. Running times for all computations are < 1 hour, with a big majority being < 30 min.

#### Longest archaic fragment

The time complexity of this algorithm is

$O\left( m\left( s+\sum_{s}^{l} k \right)^{2} \right)$ =$O\left( m\left( s+(l-s+1)\frac{(l+s)}{2} \right)^{2} \right)$.

In the worst-case scenario, $s = 1$ we get

$O\left( m\left( \frac{l^{2}}{2} \right)^{2} \right) \approx$ $O\left( ml^{4} \right)$,

where $m$ is the sequence length (genome length, 10^6^ for simulations), $s$ and $l$ are the minimum (260 in simulations) and maximum (380 in simulations) archaic fragment length, respectively, for which a probability will be computed and for all lengths in between. The first strategy to reduce computational time is to minimise the distance between $s$ and $l$. We set $s$ to 260 and $l$ to 380 by approximating the corresponding values from the samples from the posterior (Figure 3). The second strategy is to compute sections of the distribution independently. For example, if we compute sections of length $z$, the first section would compute from $s$ to $s+z$, and the second $s+z$ to $s + 2z$, and so on. The resulting sections can be aggregated together to form the probability distribution $s$ to $l$. We set $z$ to 5 in the simulation study. Running times for all computations are < 4 hours.

### S3 - Robustness to model misspecifications

We test the robustness of the sampling from the posterior approach to misspecification and violations of the following HMM assumptions:

1. Weak contrast of emission rates between states. This corresponds to situations where the coalescent times between Africans and Non-Africans are closer to coalescent times between archaic and modern humans.
2. Overdispersed observations (non-Poisson counts). This corresponds to coalescent events of a given state not happening at the same time point across the genome, but having some variation, inflating variance relative to the Poisson assumption.
3. Non-geometric fragment lengths. This can arise under non-uniform recombination or multiple admixture events, both of which deviate from the geometric length distribution implied by a homogeneous HMM.
4. Imperfect removal of variants shared with the outgroup. Limited outgroup sampling and demographic history can leave “common” variants in the data, adding noise to both states and reducing power.

A helpful analogy to the inhomogeneous sampling process is Bayesian statistics. One can think of the set of parameters $\pi$, $\Gamma$ and $\Phi$ as the priors, while the observations are the data. The sampling approach samples the posterior distribution of the hidden state sequences. When the data are highly informative (e.g., emission rates differ strongly between states), the posterior is dominated by the likelihood; when the signal is weak, the posterior resembles the prior.

In this section, we focus on the ability to capture the true summary statistics such as fragment length distribution, number of fragments, total sequence, accuracy and precision. Testing the sampling approach will, by extension, also serve as a test of the FMCI.

#### Differentiation of emission parameters

We simulate data with realistic parameters (Table 1). We vary $\lambda_{A}$ from the original realistic parameters to the values 0.031, 0.06, 0.09, 0.12, 0.15, 0.3, 1, 2, 3 to evaluate how robust the inhomogeneous method is to the difference between the emission parameters in the two states.

We simulate 10^6^ windows of sequence. For each simulated dataset, we obtain 100 samples from the posterior with both the known parameters (the parameters which generated the data) and the estimated parameters obtained from Baum Welch training. We also decode fragments using Viterbi and Posterior decoding under the same conditions. We obtain these simulations with hmmix make_test_data. We show the results in Figure S3.

When we provide the inhomogeneous sampling approach with the known parameters, we obtain distributions of the summary statistics that overlap the true values, no matter how differentiated the emission parameters $\lambda_{A}$ and $\lambda_{H}$ are. This is because we are always providing the correct prior. Thus, even when there is no signal in the data (e.g., $\lambda_{A}$ = 0.031), and fragments are incorrectly inferred (accuracy and precision close to 0%), summary statistics are well captured. This behaviour is not observed with the Posterior decoding and Viterbi methods, which focus on the maximum likelihood estimate.

In general, when parameters are estimated instead of directly provided (right panels), $\lambda_{A}$ are within 2% of the true parameter value, which leads to well-estimated summary statistics by the inhomogeneous sampling method. The exceptions are when true $\lambda_{A}$ is 0.031 and 0.06 (very similar to $\lambda_{H}$ which is 0.03), and thus, there is not enough differentiation between emission probabilities between states and thus, not enough power. Here, the trained $\lambda_{A}$ are 0.0547 and 0.0542, respectively. The incorrect estimation of parameters means we have an incorrect prior. Since there is little signal in the data, this leads to incorrectly estimated fragments and thus summary statistics.

However, for the case of archaic admixture in modern humans, we are in a situation in which the Poisson rate from the archaic hidden state is $\sim0.3$ [(Skov et al. 2018; Skov et al. 2020)](https://app.readcube.com/library/b0a9d0bb-10df-4f1c-a9f9-fbbf1e9e85ec/all?uuid=2706874926890608&item_ids=b0a9d0bb-10df-4f1c-a9f9-fbbf1e9e85ec:52c1bc52-d18b-4db8-baa8-383e908ef709,b0a9d0bb-10df-4f1c-a9f9-fbbf1e9e85ec:761d10b0-f37f-4911-8a21-34132f010854), and for that regime, sampling from the posterior shows robustness to recover the right summary statistics in this idealistic simulated case, outperforming Viterbi and Posterior decoding. In that range, decodings have accuracy and precision values above 85%. We also observe that accuracy is lower for the sampling approach than for the other two methods. This is because fragments with low posterior probability are also represented in the sampling approach, thus having a greater chance of being false positives, reducing accuracy and precision compared to Posterior decoding.


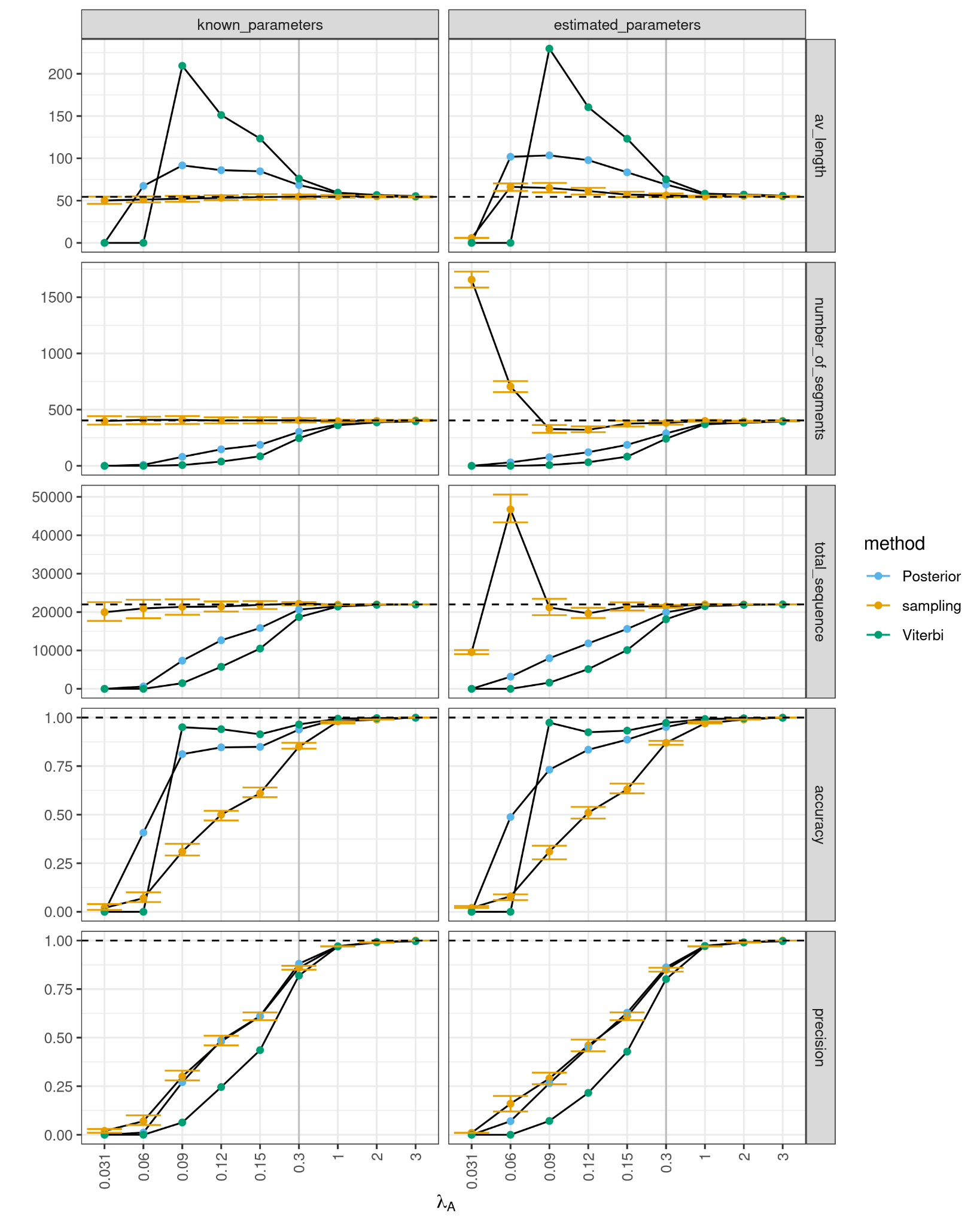


##### Figure S3. The dotted horizontal line indicates the true simulated values for each summary statistic shown in horizontal panels. We show the effect of varying $\lambda_{A}$ for 100 samples from the posterior, the Posterior decoding and Viterbi, where we use the true parameters (left panels) and the trained parameters (right panels). The grey vertical line shows the realistic archaic emission parameter as shown in the literature studying archaic introgression in present-day humans.

#### Observation generated from an overdispersed Poisson process

hmmix assumes that observations are generated from a Poisson process - this corresponds to assuming that all fragments coalesce at a single time point. However, under coalescence theory, coalescence is expected to be exponentially distributed. We now test the robustness of the sampling approach when the observations are generated from an overdispersed distribution.

For this test, we simulate using the realistic parameters (Table 1), but now generate observations in human state, archaic state or both the human and archaic state from a negative binomial distribution - instead of a Poisson distribution - where the mean is 0.03 and 0.3, respectively, but the variance changes. We express the overdispersion as the ratio of the variance to the mean.

We simulate 10^6^ windows of sequence. For each simulated dataset, we obtain 100 samples from the posterior with both the known parameters (the parameters which generated the data) and the estimated parameters obtained from Baum Welch training. We also decode fragments using Viterbi and Posterior decoding under the same conditions. We obtain these simulations by modifying the base code of hmmix make_test_data to sample observations from a negative binomial distribution from the provided parameters. We show the results in Figure S4. The first simulation ratio of 1, which is equivalent to generating observations from a Poisson distribution. We next increase the ratio to 1.03125, 1.0625, 1.125, 1.25 and 1.5. We show the results in Figure S4.

The sampling approach fails to obtain the true summary statistics when the variance/mean ratio is equal to or greater than 1.125 for the human state or both states. This is true whether or not we use the known or estimated parameters. This can be explained by the signal in the data (the overdispersion) having a larger impact than the prior. We note that overdispersion in the archaic state has a minimal impact on the summary statistics. This is because overdispersion in the archaic state will only make archaic fragments more SNP dense than the human fragments compared to the Poisson case, increasing power.

To estimate the extent of variance in real data, we calculate it in the three individuals from the 1000 Genomes Project dataset used in the main text (Table S3). To calculate the variance, we modify the base code of hmmix train to output not only the mean value of the emission parameter (used as the estimated emission parameters), but also the variance. We observe that the ratio variance/mean never exceeds 1.2 for the Archaic state and 1.03 for the Human state. These realistic estimates are shown as vertical dotted lines in Figure S4. We observe that in this regime, parameters are well estimated by the sampling from the posterior approach.


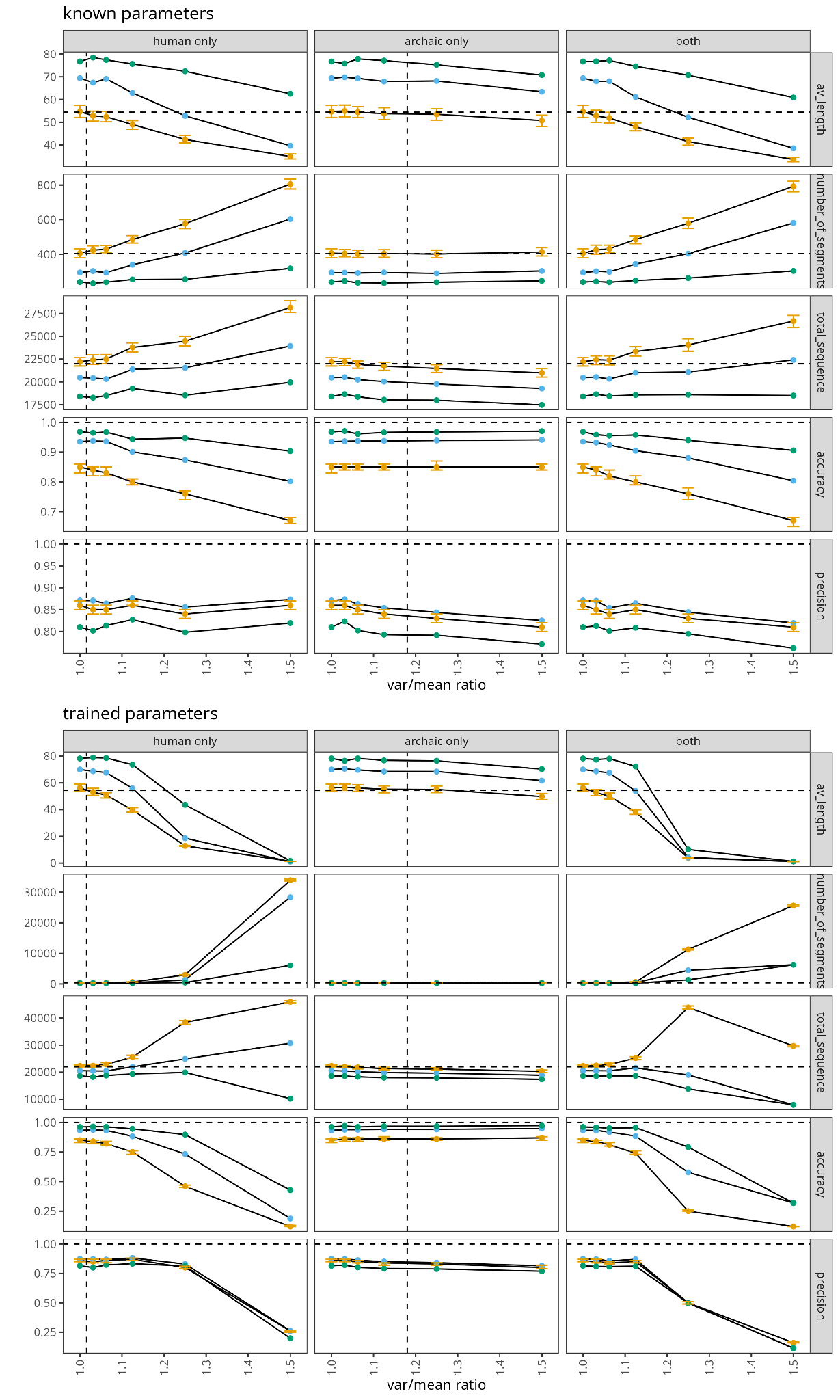


##### Figure S4. The dotted horizontal line indicates the true simulated values for each summary statistic shown in horizontal panels. The dotted vertical line in the human and archaic state denotes the observed values in three individuals from the 1000 Genomes Project dataset (Table S3). We show the effect of increasing the variance of the negative binomial distribution compared to the mean for 100 samples, where we use the true parameters and the trained parameters.

##### Table S3. Emission parameters and their variance for human and archaic state for three 1000 Genomes Project individuals for both haploid and diploid genomes.

| **Phased** | **Individual** | **State** | **Emission** | **Variance** | **Ratio**  **(variance/mean)** |
| --- | --- | --- | --- | --- | --- |
| Haploid | NA20810  (TSI European) | Human | 0.0201 | 0.0204 | 1.0168 |
|  |  | Archaic | 0.3177 | 0.3779 | 1.1893 |
|  | NA21130  (GIH South Asian) | Human | 0.0231 | 0.0235 | 1.0191 |
|  |  | Archaic | 0.3251 | 0.3920 | 1.2061 |
|  | NA19078  (JPT East Asian) | Human | 0.0221 | 0.0225 | 1.0183 |
|  |  | Archaic | 0.3217 | 0.3857 | 1.1988 |
|  | | | | | |
| Diploid | NA20810  (TSI European) | Human | 0.0390 | 0.0399 | 1.0234 |
|  |  | Archaic | 0.3550 | 0.4203 | 1.1838 |
|  | NA21130  (GIH South Asian) | Human | 0.0446 | 0.0458 | 1.0272 |
|  |  | Archaic | 0.3641 | 0.4375 | 1.2015 |
|  | NA19078  (JPT East Asian) | Human | 0.0417 | 0.0428 | 1.0254 |
|  |  | Archaic | 0.3597 | 0.4332 | 1.2046 |

We also investigate how demographic parameters impact the variance/mean ratio with simulations. Recall that the emission parameter is related to the minimum coalescence time between the ingroup and the outgroup. The minimum coalescence time with the outgroup follows an exponential distribution with a parameter that depends on the effective population size and number of samples from the outgroup. Thus, increasing the effective population size of the ancestral population will increase the mean coalescence time. Since coalescent events are exponentially distributed, the variance will increase more rapidly than the mean, thus increasing the ratio variance/mean.

In this case, we only focus on the emission parameter for the human state as it has the biggest impact on recovering the right summary statistics (Figure S4) and set the effective population size of the ancestral population of Africans and non-Africans to 1,000, 10,000, 25,000 and 50,000 individuals. We name these simulation scenarios 1-4, respectively (Table S4).

For each scenario, we simulate with msprime [(Baumdicker et al. 2021)](https://app.readcube.com/library/b0a9d0bb-10df-4f1c-a9f9-fbbf1e9e85ec/all?uuid=2777423738213658&item_ids=b0a9d0bb-10df-4f1c-a9f9-fbbf1e9e85ec:35016448-51ea-4d4e-9f25-c968566a05dc) a non-African genome and 500 African genomes from a simple demography with the following parameters:

The non-African and African populations split 50,000 years ago. The effective population size of Africans after the split is set to 20,000 individuals. The non-African population goes through a bottleneck where the effective population size reduces to 1,000 individuals in the time interval 45,000 - 50,000. After that, it recovers to 10,000 individuals. The YAML files, along with scripts for simulating the scenarios, are provided in the GitHub folder (<https://github.com/MoiColl/HMMenhancements>).

For each scenario, we simulate 10,000 independent 1 kb windows and count the number of variants which are not seen in the 500 African genomes. We calculate the average number of SNPs per 1 kb window, i.e. the emission parameter for the human state. We also calculate the ratio of the variance/mean. We show the results in Table S4.

##### Table S4. Simulation parameters for increasing the variance-to-mean ratio of the emission rates.

|  | Anc pop Ne | Emission parameter | Ratio (var/mean) |
| --- | --- | --- | --- |
| Scenario 1 | 1,000 | 0.0241 | 1.001 |
| Scenario 2 | 10,000 | 0.0364 | 1.002 |
| Scenario 3 | 25,000 | 0.0513 | 1.038 |
| Scenario 4 | 50,000 | 0.0788 | 1.068 |

It is often assumed that the effective population size of humans is around 20,000 (<https://popsim-consortium.github.io/stdpopsim-docs/stable/catalog.html#sec_catalog_homsap_models_outofafricaextendedneandertaladmixturepulse_3i21>)[(Lauterbur et al. 2023)](https://app.readcube.com/library/b0a9d0bb-10df-4f1c-a9f9-fbbf1e9e85ec/all?uuid=23778895425388702&item_ids=b0a9d0bb-10df-4f1c-a9f9-fbbf1e9e85ec:e2995fbd-acf0-4f29-b64f-d5010921d7b3). If we assume an effective population size of non-African/African is between 10,000 - 25,000 individuals, we expect an emission parameter around 0.03 and a low degree of overdispersion (variance/mean ratio between 1.002 and 1.038). Thus, we expect that archaic introgression summary statistics will be well recovered with the sampling from the posterior approach, with the level of coalescent overdispersion given realistic demographic parameters. Even if the effective population size of non-African/Africans was up to 50,000, the ratio would be in a regime where the summary statistics are well estimated (Figure S4).

#### Fragment length distribution of archaic states generated from a non-geometric distribution

Sampling from a homogeneous HMM produces a fragment length distribution that is geometric. For example, the length distribution of a given archaic state $A$ is geometrically distributed with the rate equal to the probability of leaving the state (${1-\Gamma}_{AA}$). This is true not only for archaic introgression analysis here, but for HMMs in general.

Thus, in the absence of any signal in the data differentiating the two states, the prior distribution will dominate the posterior and sampling from an inhomogeneous HMM will then produce a geometric distribution. Here, we investigate the flexibility of the inhomogeneous sampling approach to recover non-geometric distributions compared to the Posterior decoding and Viterbi algorithm.

We first test the sampling approach to capture a fragment length distribution defined by a combination of a Poisson distribution and a geometric distribution. We also test the performance of the sampling approach in the presence of a varying recombination rate, which will tend to skew the distribution towards shorter fragments. Finally, we test a scenario of multiple admixture events, which corresponds to a mixture of multiple geometric distributions related to each gene-flow event.

##### Mixture of geometric and Poisson distributions

We simulate 10^6^ windows of data from the realistic parameters (Table 1) varying $\lambda_{A}$ = 0.06, 0.3 and 3 and under two scenarios.

1. In scenario 1, 75% of archaic fragments follow a geometric distribution with parameter ${1 - \Gamma}_{AA}$ and 25% of archaic fragments follow a Poisson distribution with mean = 50.
2. In scenario 2, 100% of the archaic fragments are generated from a Poisson distribution with mean=50.

We obtain these simulations by modifying the base code of hmmix make_test_data to create archaic fragments such that their length is sampled from the distributions of the two scenarios described above. We show the results in Figure S5.

When the emission parameters are very similar between the human and the archaic state ($\lambda_{A}$ = 0.06 and $\lambda_{H}$ = 0.03), there is not enough signal in the data to recover the true length distribution. The sampled length distribution is instead very similar to the geometric distribution expected from the estimated parameters (red dotted line in Figure S5), showing a strong importance of the prior probabilities over the data. When the emission parameters become sufficiently distinct ($\lambda_{A}$ = 0.3 and $\lambda_{H}$ = 0.03), the sampling approach approximates better the true length distribution and we observe a distortion to the decaying shape of the geometric distribution, spotting a peak where the Poisson distribution is centered. Finally, when the two states are highly differentiated ($\lambda_{A}$ = 3 and $\lambda_{H}$ = 0.03), the sampling approach recovers the simulated distribution, showing a strong importance of the data over the prior probabilities. Therefore, we conclude that with sufficient information in the data, sampling from the posterior is able to recover fragment length distributions that are highly different from the assumed geometric distribution.

**
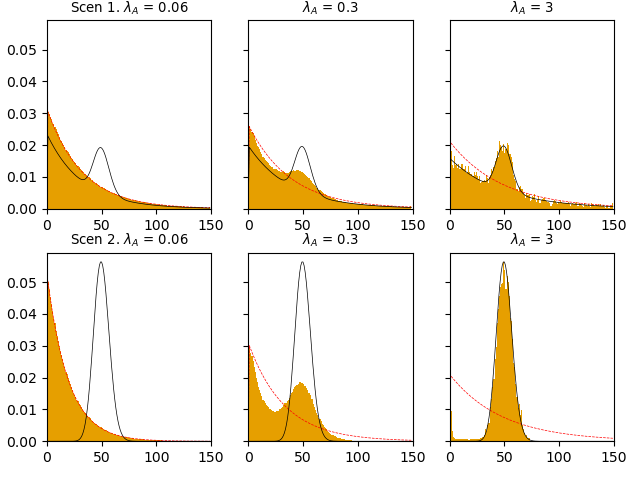
**

##### Figure S5. Length distribution from 100 samples from the data under two different scenarios and with three different emission values for the archaic state. The black line shows the true length distribution, the red line shows the geometric distribution with a parameter equal to the estimated transition parameter from the archaic state (${1 - \Gamma}_{AA}$), and the histogram is the length distribution from the 100 samples from the posterior.

##### Recombination map

We next investigate if the inhomogeneous sampling approach can capture the archaic fragment length that is generated by a constant and varying recombination rate across the genome.

Using msprime [(Baumdicker et al. 2021)](https://app.readcube.com/library/b0a9d0bb-10df-4f1c-a9f9-fbbf1e9e85ec/all?uuid=7790089648178472&item_ids=b0a9d0bb-10df-4f1c-a9f9-fbbf1e9e85ec:35016448-51ea-4d4e-9f25-c968566a05dc), we simulate a complete genome of 2.8 Gb with constant and varying recombination rates. We extracted true archaic introgressed fragments and added SNPs to the genome using $\lambda_{H}$ = 0.03 for human fragments $\lambda_{A}$ = 0.06, 0.3 and 3 for archaic fragments. We train the HMM parameters and decode the hidden state sequence using Viterbi and Posterior decoding algorithms. We also sample 100 paths from the conditional posterior probabilities. The YAML files, along with scripts for simulating the scenarios, are provided in the GitHub folder (https://github.com/MoiColl/HMMenhancements).

The results in simulations with a constant recombination rate are shown in Figure S6. For those simulations, the true fragment length distribution follows a geometric distribution, and the sampling approach recovers the correct distribution for $\lambda_{A}$ = 0.06 and $\lambda_{A}$ = 0.3. Viterbi and Posterior decoding perform worse at recovering the right fragment length distribution, with Viterbi not even recovering any fragment when $\lambda_{A}$ = 0.06 (Figure S6b). For $\lambda_{A}$ = 3, Viterbi, Posterior Decoding and the sampling approach recover the correct fragment length distribution (Figure S6b).


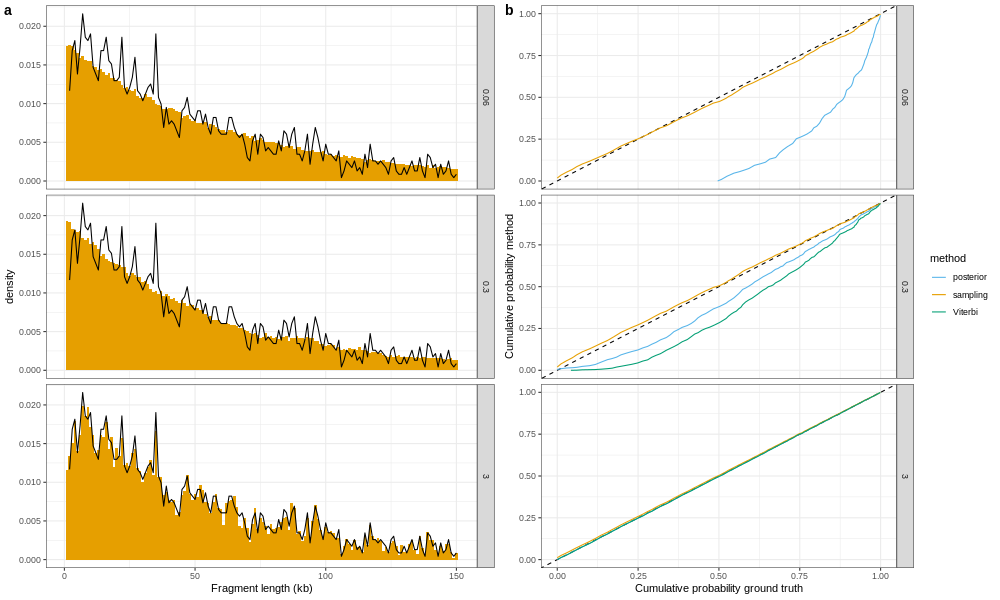


##### Figure S6. a) Fragment length distribution. True distribution is shown in black for a 2.8 Gb genome with a constant recombination rate simulated with msprime. In orange, the fragment length distribution from 100 inhomogeneous samples is shown. b) Reverse cumulative probability density function (starting from the longest fragments). Only fragments shorter than 150 kb are considered. Note that for $\lambda_{A}=0.06$ scenario, Viterbi decoding does not call any archaic fragment.

The results in simulations with varying recombination rate are shown in Figure S7. We note that the true length distribution is much more skewed towards shorter fragments than in the constant recombination rate scenario. Sampling from the posterior, as well as Posterior decoding and Viterbi, misses a big portion of short fragments for scenarios when $\lambda_{A}=0.06$ and $\lambda_{A}=0.3$. This is partially explained by many short fragments having none or very few SNPs, which makes those fragments impossible to detect. For example, 653/1839 (36%) of archaic fragments have 0 SNPs for the $\lambda_{A}=0.06$ scenario and 171/1839 (9%) for the $\lambda_{A}=0.3$ scenario. Therefore, sampling from the posterior, even though performing better than Viterbi and Posterior decoding, misses lots of short fragments in a scenario compatible with Neanderthal-human introgression ($\lambda_{A}=0.3$).

With sufficient information ($\lambda_{A}=3$), all methods recover the right fragment length distribution. In that case, 0/1839 fragments have 0 derived SNPs.

###
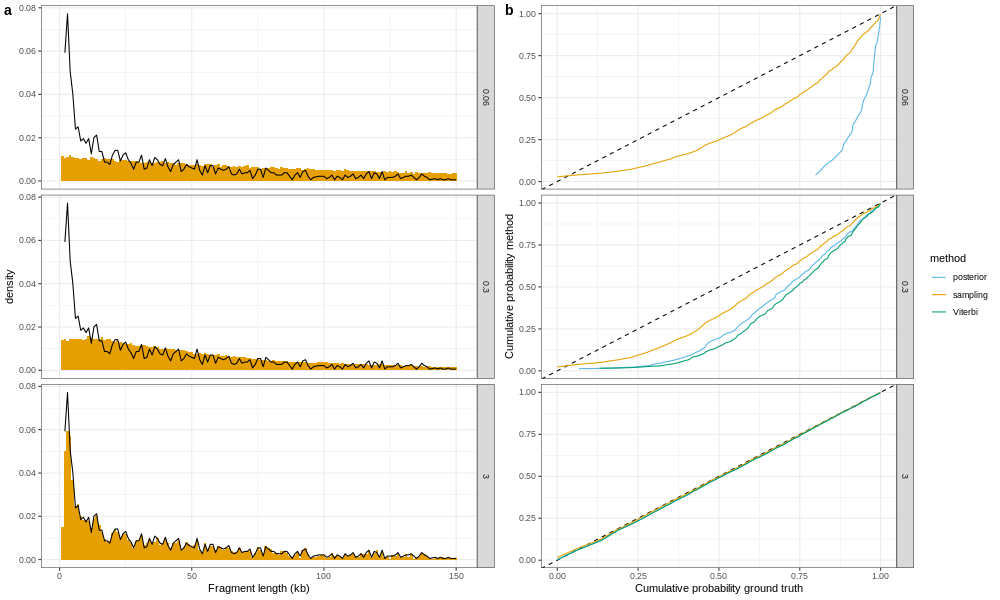


##### Figure S7. a) Fragment length distribution. True distribution is shown in black for a 2.8 Gb genome with varying recombination rate simulated with msprime. In orange, the fragment length distribution from 100 inhomogeneous samples is shown. b) Reverse cumulative probability density function (starting from the longest fragments). Only fragments shorter than 150 kb are considered.

##### Multiple admixture pulses

We investigate the effect of multiple admixture events, which generate a mixture of fragments with lengths proportional to the time since admixture of the corresponding pulse, thereby causing the final length distribution to deviate from a single geometric distribution. We simulate 2.8 Gb of sequence in [(Baumdicker et al. 2021)](https://app.readcube.com/library/b0a9d0bb-10df-4f1c-a9f9-fbbf1e9e85ec/all?uuid=13795482735141484&item_ids=b0a9d0bb-10df-4f1c-a9f9-fbbf1e9e85ec:35016448-51ea-4d4e-9f25-c968566a05dc) under a constant recombination rate with two admixture events occurring at 45,000 years ago and 20,000 years ago, respectively. Both contribute 2.5% to the genome (a total archaic admixture of 5%). The YAML files, along with scripts for simulating the scenarios, are provided in the GitHub folder (https://github.com/MoiColl/HMMenhancements).

The results are shown in Figure S8. We note that the sampling procedure can effectively recover the length distribution of multiple admixture events in the case of a constant recombination rate and emission values which are similar to archaic/human introgression ($\lambda_{A}=0.3$).


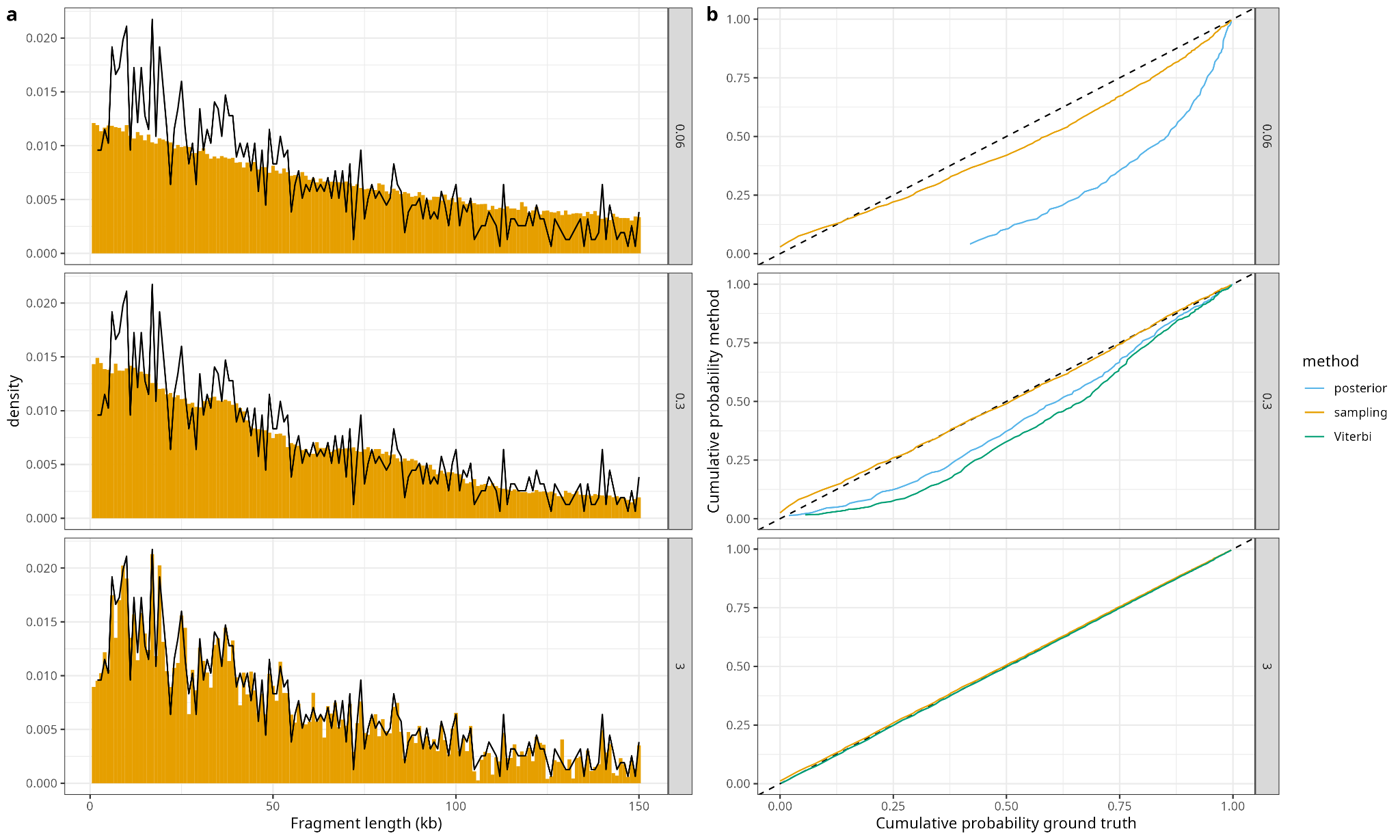


##### Figure S8. a) Fragment length distribution. True distribution is shown in black for a 2.8 Gb genome with two admixture events occurring at 45,000 years ago and 20,000 years ago with msprime. In orange, the fragment length distribution from 100 inhomogeneous samples is shown. b) Reverse cumulative probability density function (starting from the longest fragments). Only fragments shorter than 150 kb are considered.

#### Ability to remove common variation with an outgroup sample

It may not always be possible to remove all common variants between the ingroup and the outgroup - i.e., variants that arose in their common ancestor (Figure 1 of the main text). This can happen if only a limited number of outgroup genomes are available, or if outgroup lineages coalesce rapidly due to their demographic history. Either case introduces noise into both the Human and Archaic states, reducing hmmix’s ability to distinguish them. Here, we investigate the consequences of inefficient filtering of common variants driven by these factors.

We first simulate 100 Mb of sequence from a simple demography (Demography a) in Figure S10) using msprime [(Baumdicker et al. 2021)](https://app.readcube.com/library/b0a9d0bb-10df-4f1c-a9f9-fbbf1e9e85ec/all?uuid=07960699144506078&item_ids=b0a9d0bb-10df-4f1c-a9f9-fbbf1e9e85ec:35016448-51ea-4d4e-9f25-c968566a05dc). For derived variants in the ingroup genome, we track whether it is private to the ingroup population (“private”) or arises in the ingroup/outgroup ancestor (“common”). We generate observed data for hmmix by keeping all private variants and removing between 80-100% of the common variants. We train the model parameters and estimate the summary statistics with Posterior decoding, Viterbi and the sampling approach. The YAML files, along with scripts for simulating the scenarios, are provided in the GitHub folder (https://github.com/MoiColl/HMMenhancements).

Figure S9 shows that removing >95% of common variants results in well-estimated summary statistics by the sampling approach, while Viterbi and Posterior decoding estimates are biased even when common variant filtering is perfect. This is in line with the main text results.


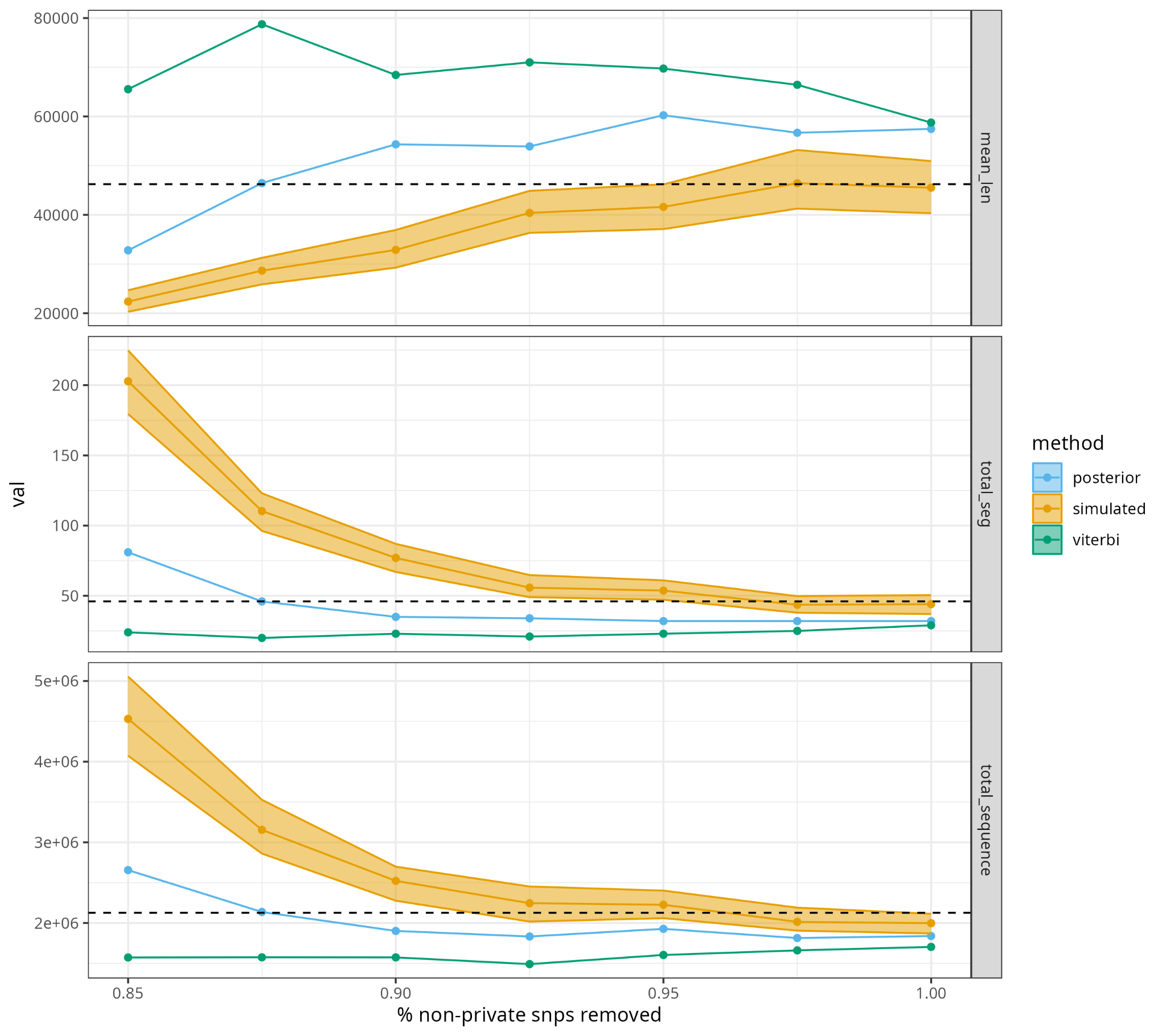


##### Figure S9. Estimated summary statistics as a function of common variants removed. The error bars represent 95% percentile of the summary statistics for 50 samples from the posterior.

We next investigate how the outgroup sample size affects the fraction of common variants removed. We simulate 100,000 windows of 1kb for eight different demographic scenarios that vary key parameters (Figure S10). We also compare scenarios where the outgroup consists of one or three populations to mimic the human-archaic introgression scenario, in which the outgroup individuals belong to three African populations - YRI, MDL, ESN - from the 1000 Genomes Project, thus mirroring population structure.

The eight demographies we simulate from are:

a) Standard demography, where Ne for all populations is 10kb

b) Increase Ne to 50k for the ancestral population of ingroup/outgroup(s).

c) Add exponential growth to the 3 outgroup populations, increasing their Ne to 50k

d) Outgroup coalesce order

e) Increase Ne of all outgroups to 50k, keeping the size of the ingroup and the ingroup/outgroup common ancestor at 10k

f) Same as e) but increasing Ne of all outgroups to 100k

g) Add symmetric migration rates between all outgroup populations at 2e-4 migrants per generation and migration from ingroup to the outgroup of 1.7e-5 migrants per generation. This corresponds to the scenario called the low back-migration rate scenario from a previous publication [(Identifying and Interpreting Apparent...)](https://paperpile.com/c/UzacFy/CtaJ)

h) Same as g) but simulating a high back-migration rate (rates 2e-4 and 5e-4, respectively)

Table S5 reports the number of common and private variants for each scenario, as well as the emission parameters and the variance/mean ratio of the emission parameters.


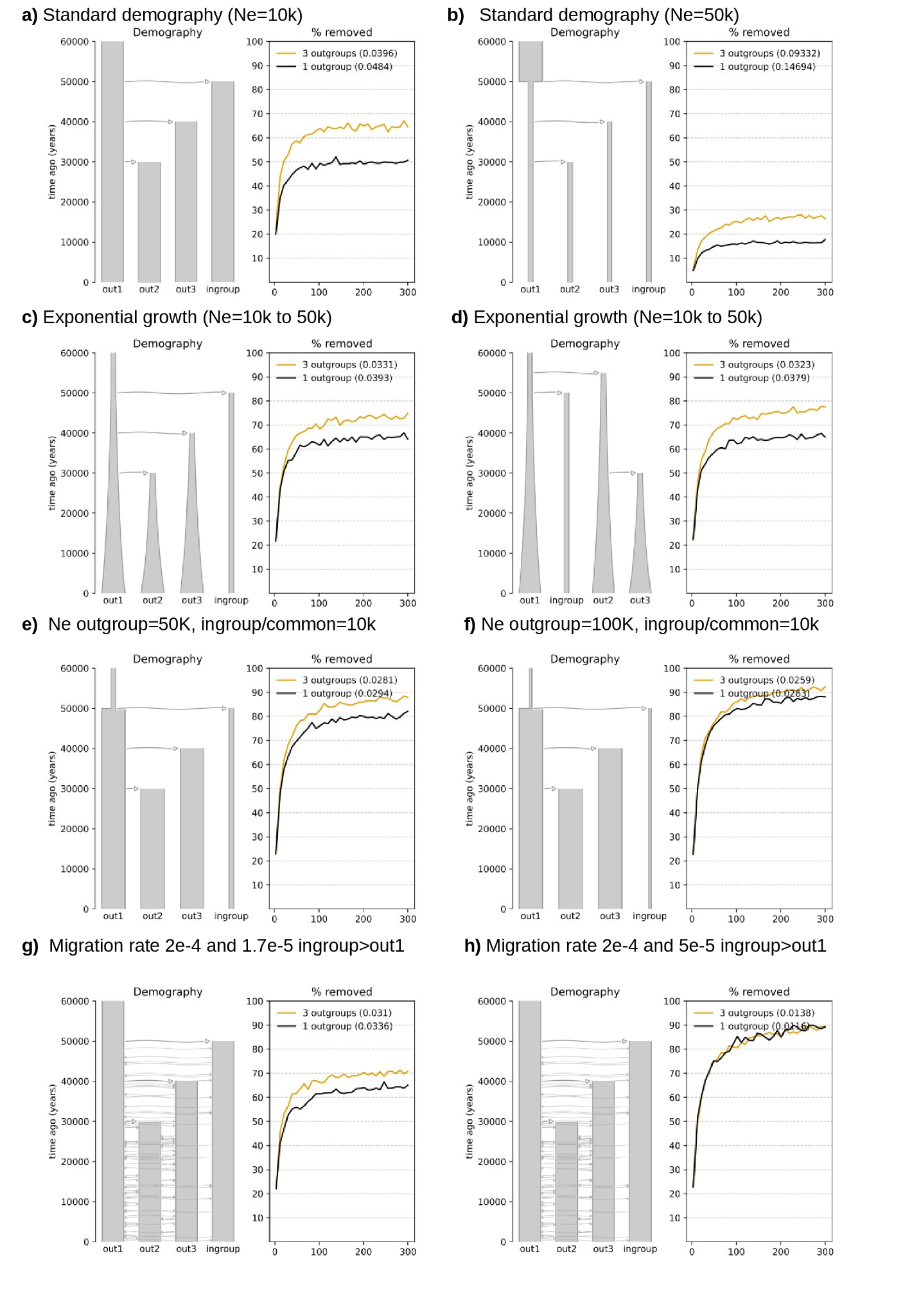


##### Figure S10. Effectiveness of removing common variants as a function of outgroup size for outgroups consisting of a single population (out1 population only, black) or three populations (out1, out2, and out3 populations, orange). For each scenario, the number of private variants per 1kb is shown, which is equivalent to the emission parameter for the ingroup state in hmmix.

##### Table S5. Variants removed, emission parameters and the variance/mean ratio of the emission parameter for the eight simulated scenarios.

| Scenario | n pops | Common var | Private var | Removed (%) | emission | mean/var |
| --- | --- | --- | --- | --- | --- | --- |
| a) Standard demography (Ne=10k) | 1 pop | 2,545 | 2,558 | 50.13 | 0.051 | 1.12 |
|  | 3 pop | 1,390 | 2,547 | 64.69 | 0.039 | 1.08 |
| b) Standard demography (Ne=50k) | 1 pop | 12,093 | 2,601 | 17.70 | 0.150 | 1.63 |
|  | 3 pop | 6,871 | 2,461 | 26.37 | 0.090 | 1.47 |
| c) Exponential growth (Ne=10k to 50k) | 1 pop | 1,447 | 2,470 | 63.06 | 0.039 | 1.09 |
|  | 3 pop | 863 | 2,467 | 74.08 | 0.033 | 1.05 |
| d) Exponential growth (Ne=10k to 50k) pop flipped | 1 pop | 1,317 | 2,557 | 66.00 | 0.039 | 1.06 |
|  | 3 pop | 737 | 2,452 | 76.89 | 0.032 | 1.03 |
| e) Ne outgroup=50K, ingroup/common=10k | 1 pop | 571 | 2,448 | 81.09 | 0.030 | 1.04 |
|  | 3 pop | 340 | 2,501 | 88.03 | 0.028 | 1.03 |
| f) Ne outgroup=100K, ingroup/common=10k | 1 pop | 302 | 2,490 | 89.18 | 0.028 | 1.03 |
|  | 3 pop | 221 | 2,430 | 91.66 | 0.027 | 1.03 |
| g) Migration rate 2e-4 and 1.7e-5 ingroup>out1 | 1 pop | 1,148 | 2,150 | 65.19 | 0.033 | 1.07 |
|  | 3 pop | 865 | 2,175 | 71.55 | 0.030 | 1.05 |
| h) Migration rate 2e-4 and 5e-5 ingroup>out1 | 1 pop | 127 | 1,052 | 89.23 | 0.012 | 1.02 |
|  | 3 pop | 152 | 1,328 | 89.73 | 0.015 | 1.02 |

We identify scenarios f) and e) - structured outgroup with relatively high Ne values, while keeping the common ancestor and the ingroup with relatively low Ne values - as the cases which produce the most similar Human emission value (0.0259 and 0.0281, respectively) to the average value of the three non-African individuals from the 1000 Genomes Project (0.0236, Table S3). The scenarios with the least similar emission values are scenario b) (0.0933), where too many variants remain, inflating the emission parameter, and scenario h) (0.0116), where too few variants remain due to a high migration rate between the ingroup and outgroup.

We then compute the summary statistics—mean fragment length, total archaic sequence, and number of archaic fragments— to evaluate performance in estimating those statistics under different demographic scenarios. We also examine how the outgroup sample size (30,150, 300, 600) impacts the estimates, and compare to an idealised case in which all common variants are removed (“perfect”). Results are shown in Figure S11.

Under perfect filtering, sampling from the posterior recovers all three summary statistics, while Posterior decoding and Viterbi remain biased, consistent with the main-text HMM simulations (Figures 3–4). The effect of outgroup sampling depends strongly on the underlying demography. In scenarios e) and f)—which yield human-state emission parameters closest to real data (Figure S10)—the sampling approach recovers all three statistics once outgroup sample size exceeds 150 individuals, and it consistently outperforms Viterbi and Posterior decoding, which remain biased in the same direction as in the perfect-filtering case.


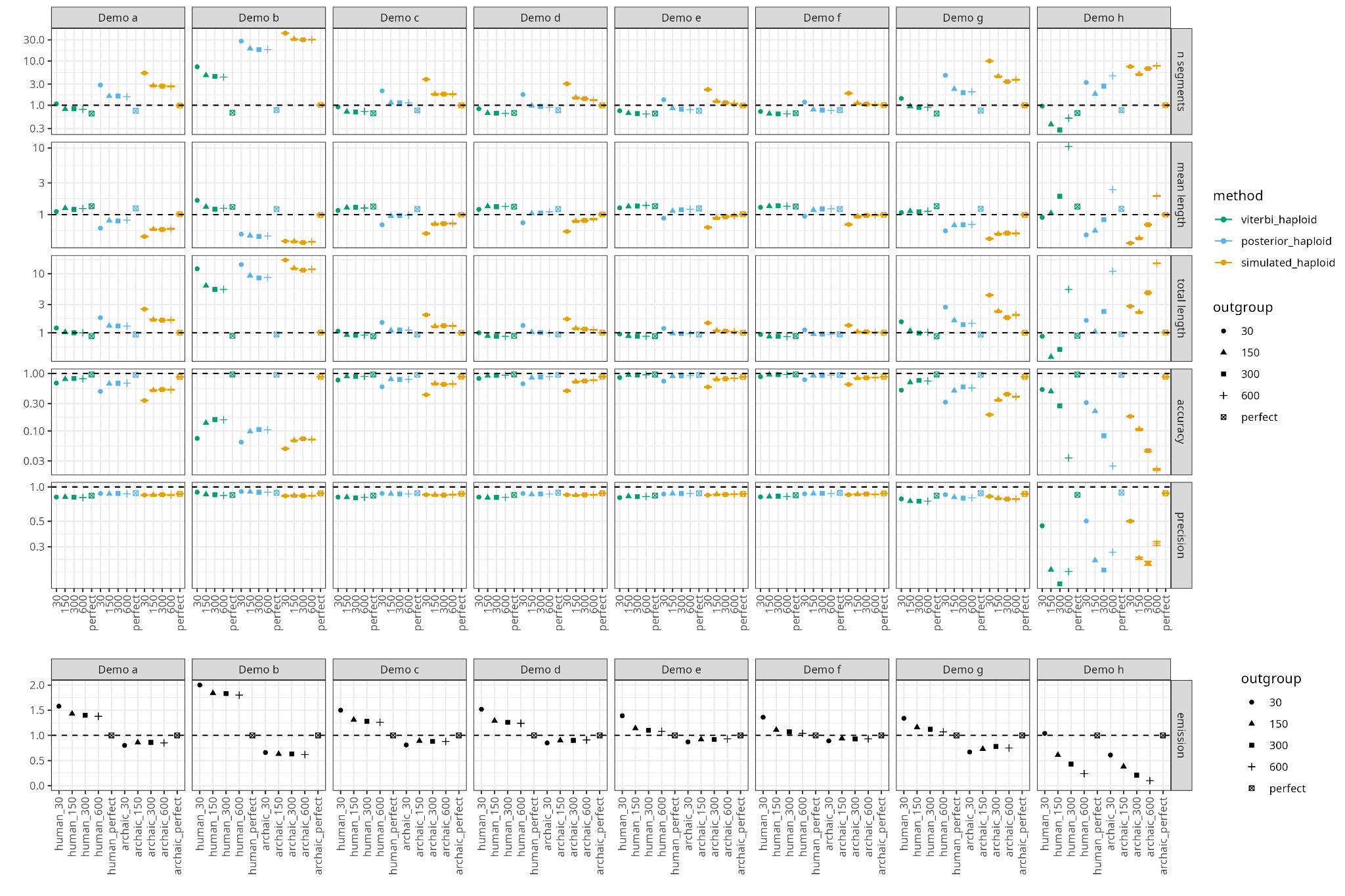


##### Figure S11. Archaic summary statistic estimation across eight simulated demographic scenarios with a constant recombination rate. The y-axis shows the logarithm of the estimated summary statistic divided by the true value. Methods are colour-coded, and outgroup sizes are shape-coded; “perfect” denotes complete removal of common variants. The bottom panel shows estimated/true emission-parameter ratios for the Human and Archaic states.

We repeated the same analysis under a varying recombination rate (Figure S12). Performance decreases for all parameter combinations relative to constant recombination (Figure S11), consistent with the recombination-map results in the fragment-length section (Figure S7). In particular, the sampling approach tends to underestimate fragment number and overestimate mean fragment length because varying recombination generates very short fragments with few or no SNPs.

#####
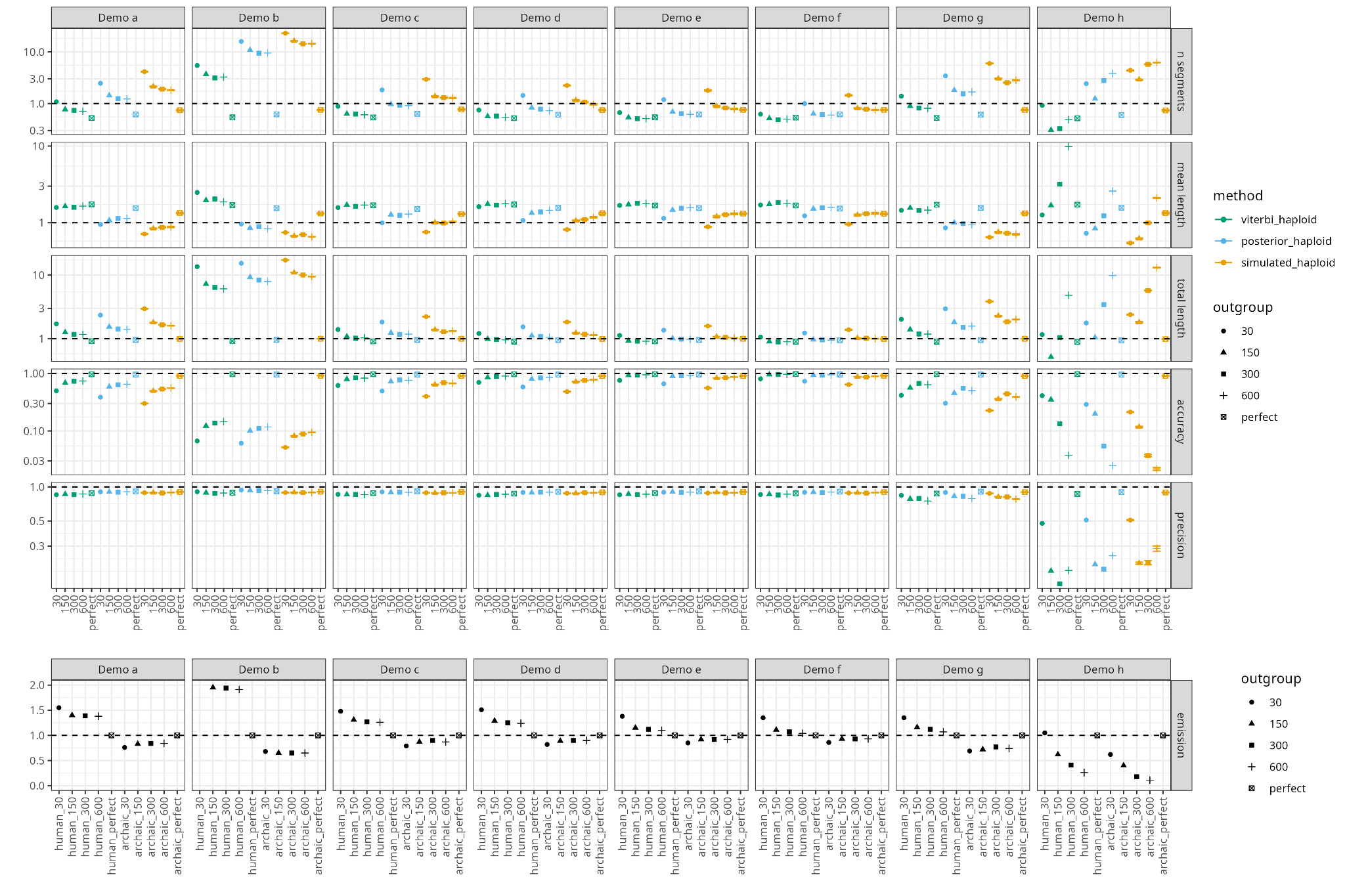


##### Figure S12. Archaic summary statistic estimation across eight simulated demographic scenarios with varying recombination rate. The y-axis shows the logarithm of the estimated summary statistic divided by the true value. Methods are colour-coded, and outgroup sizes are shape-coded; “perfect” denotes complete removal of common variants. The bottom panel shows estimated/true emission-parameter ratios for the Human and Archaic states.

Up to this point, we focused on hypothetical demographic scenarios to isolate how outgroup demography and sampling affect hmmix’s ability to recover archaic summary statistics. We next tested a more realistic scenario from [(Jacobs et al. 2019)](https://app.readcube.com/library/b0a9d0bb-10df-4f1c-a9f9-fbbf1e9e85ec/all?uuid=317795202235832&item_ids=b0a9d0bb-10df-4f1c-a9f9-fbbf1e9e85ec:14de09ad-8975-46bd-b061-9d6c9bae3aff), which includes multiple archaic admixture events into non-Africans, population structure among modern humans, and migration rates among modern human groups. We obtained demographic parameters from the stdpopsim catalogue [(Adrion et al. 2020)](https://app.readcube.com/library/b0a9d0bb-10df-4f1c-a9f9-fbbf1e9e85ec/all?uuid=022602107169349073&item_ids=b0a9d0bb-10df-4f1c-a9f9-fbbf1e9e85ec:c47a4674-d9d8-409e-9cac-87bd8bc98e58) (<https://popsim-consortium.github.io/stdpopsim-docs/stable/catalog.html#sec_catalog_homsap_models_papuansoutofafrica_10j19>). An illustration can be checked from the cited web page.

We simulate 100,000 windows of 1kb with msprime [(Baumdicker et al. 2021)](https://app.readcube.com/library/b0a9d0bb-10df-4f1c-a9f9-fbbf1e9e85ec/all?uuid=5283867300784829&item_ids=b0a9d0bb-10df-4f1c-a9f9-fbbf1e9e85ec:35016448-51ea-4d4e-9f25-c968566a05dc), varying outgroup sample size and using either constant or variable recombination (Figure S13). The results reinforce the conclusions above: sampling from the posterior recovers the summary statistics when common-variant filtering is fully effective, and outgroup sampling is sufficient, outperforming Viterbi and Posterior decoding. The two main factors that reduce performance are small outgroup sample sizes and varying recombination; the latter primarily limits detection of very short fragments (biasing summary estimates), whereas emission-parameter estimation is affected mainly by outgroup sampling.


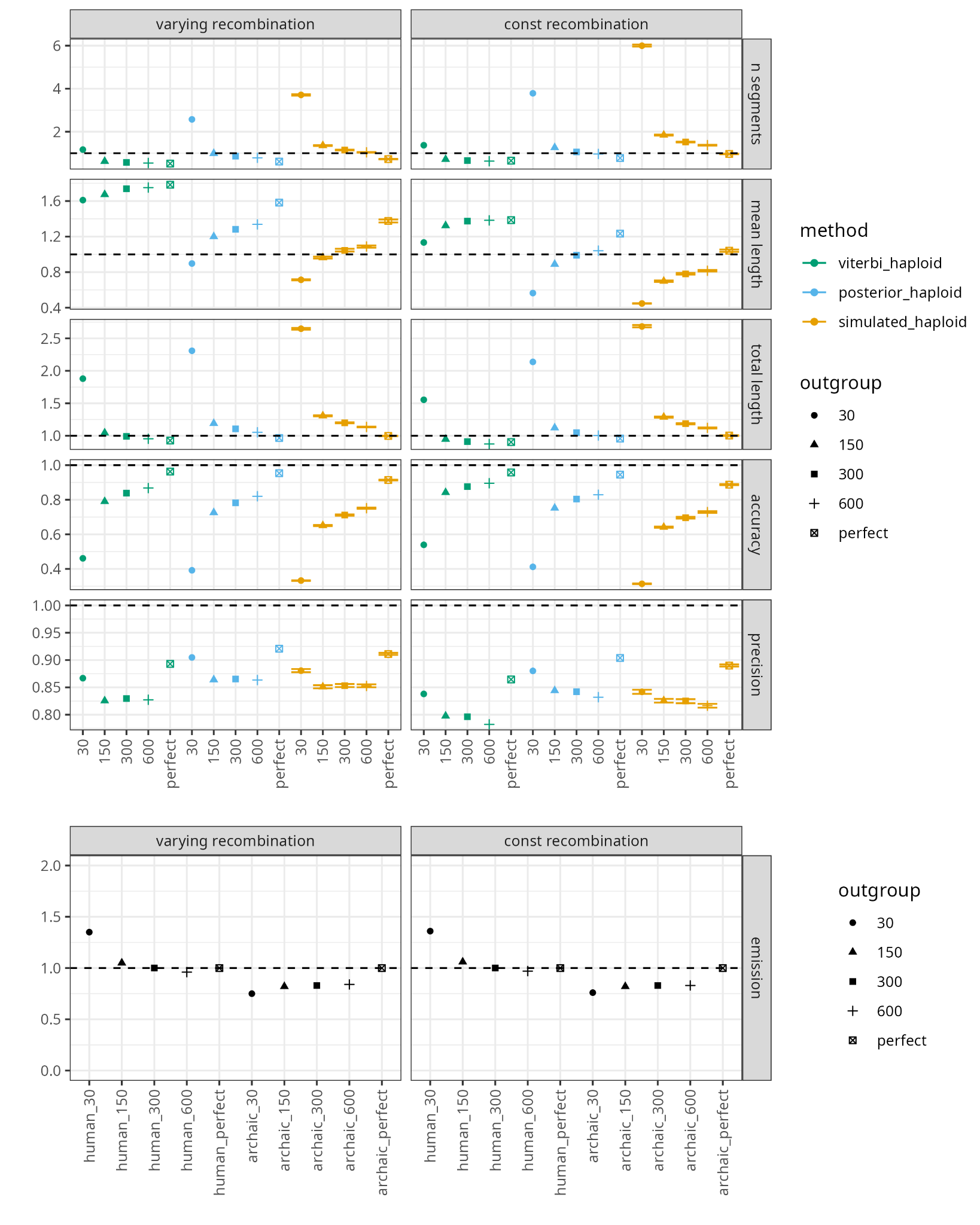


##### Figure S13. Archaic summary statistic estimation for Jacobs et al. (2019) demographic scenario with constant and varying recombination rate. The y-axis shows the logarithm of the estimated summary statistic divided by the true value. Methods are colour-coded, and outgroup sizes are shape-coded; “perfect” denotes complete removal of common variants. The bottom panel shows estimated/true emission-parameter ratios for the Human and Archaic states.

Finally, we tested whether ingroup ploidy affects performance by simulating the same model as above while sampling ingroup individuals as haploid or diploid chromosomes (constant recombination; Figure S14). We observe no substantial differences, except when the outgroup sample size is 30 individuals. We therefore conclude that hmmix recovers summary statistics similarly well from haploid and diploid data using the hmmix decode default diploid mode or the –haploid option accordingly.

#####
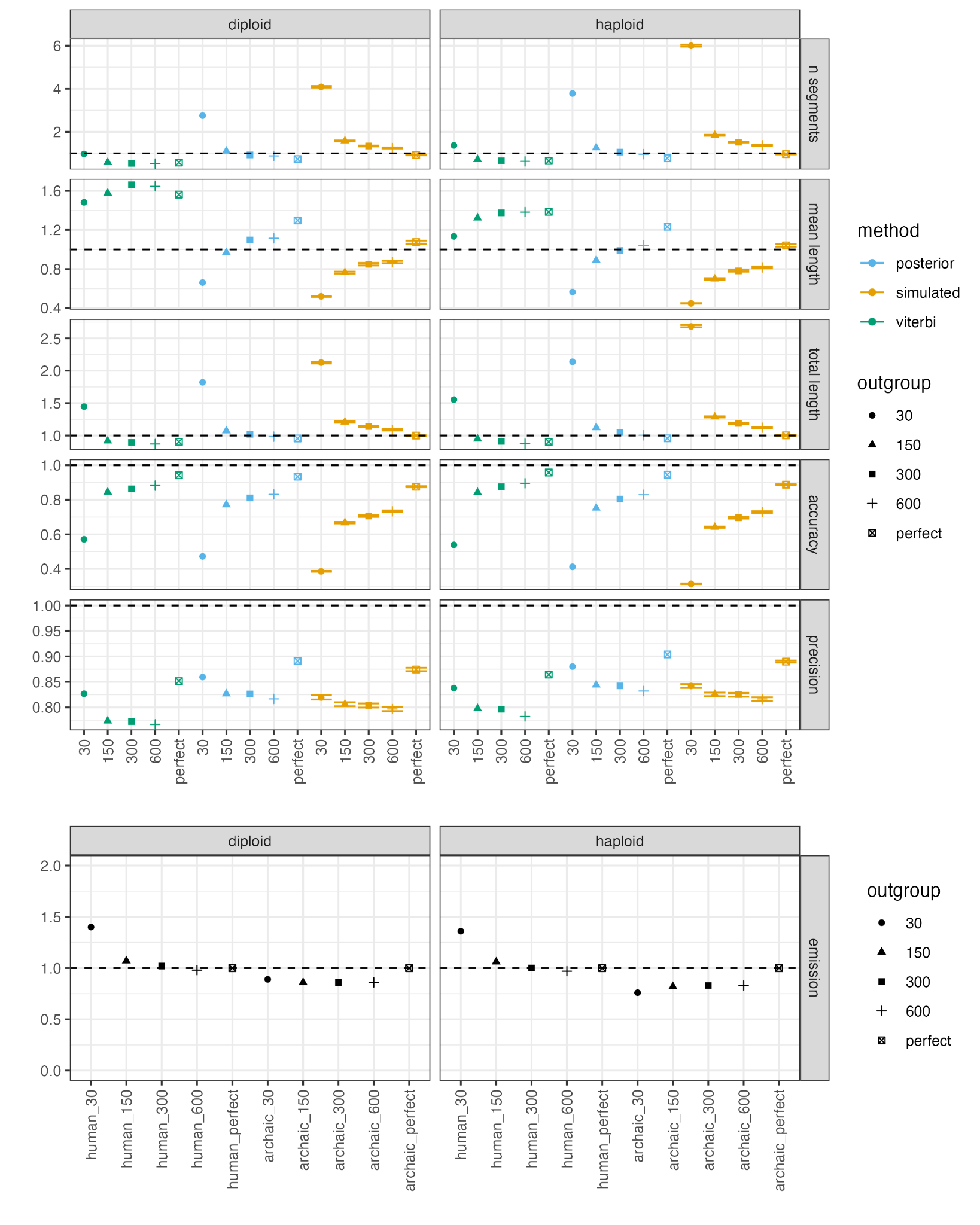


##### Figure S14. Archaic summary statistic estimation for Jacobs et al. (2019) demographic scenario with constant recombination rate for haploid and diploid data. The y-axis shows the logarithm of the estimated summary statistic divided by the true value. Methods are colour-coded, and outgroup sizes are shape-coded; “perfect” denotes complete removal of common variants. The bottom panel shows estimated/true emission-parameter ratios for the Human and Archaic states.

### S4 - Artemis statistics: log joint posterior probability and average pointwise posterior probability

The hybrid method combines the strengths of both Posterior decoding and Viterbi, balancing between the two with the $\alpha$ parameter. To tune $\alpha$, we assess the two statistics that Viterbi and Posterior decoding maximise: Log joint posterior probability and pointwise posterior probability. In this section, we show how to compute those statistics given a hidden state sequence and HMM parameters.

#### Log joint posterior probability

Viterbi maximises the posterior probability $\mathbb{P(}y_{0},...,y_{n} | x_{0},...,x_{n})$. This can be rewritten as:

$$\mathbb{P(}y_{0},...,y_{n} |x_{0},...,x_{n})=\frac{\mathbb{P(}y_{0},...,y_{n},x_{0},...,x_{n})}{\mathbb{P(}x_{0},...,x_{n})}.$$

Note that we use a zero-based-index notation and thus, $n$ corresponds to the last index in the sequence of length $m$. Thus $n = m-1$.

The likelihood of data $\mathbb{P(}x_{0},...,x_{n})$ is a constant, so maximising the posterior probability $\mathbb{P(}y_{0},...,y_{n} | x_{0},...,x_{n})$ is equivalent to maximising the joint posterior probability $\mathbb{P(}y_{0},...,y_{n},x_{0},...,x_{n})$. Furthermore, the joint posterior probability can be rewritten as:

| $\mathbb{P(}y_{0},...,y_{n},x_{0},...,x_{n})$ | $\mathbb{=P(}y_{0}\mathbb{)P(}x_{0} \vert y_{0}\mathbb{)P(}y_{1} \vert y_{0}\mathbb{)P(}x_{1} \vert y_{0},y_{1},x_{0}\mathbb{)P(}y_{2} \vert y_{0},y_{1}) ...$  $\mathbb{P(}y_{n} \vert y_{0},...,y_{n-1}\mathbb{)P(}x_{n} \vert y_{0},...,y_{n},x_{0},...,x_{n-1})$ |
| --- | --- |
|  | $\mathbb{=P(}y_{0}\mathbb{)P(}x_{0} \vert y_{0}\mathbb{)P(}y_{1} \vert y_{0}\mathbb{)P(}x_{1} \vert y_{1}\mathbb{)P(}y_{2} \vert y_{1}\mathbb{)...P(}y_{n} \vert y_{n-1}\mathbb{)P(}x_{n} \vert y_{n})$ |
|  | $\mathbb{=P(}y_{1})\prod_{t=1}^{n} \mathbb{P(}y_{t} \vert y_{t-1})\prod_{t=0}^{n} \mathbb{P(}x_{t} \vert y_{t})$ |

Because of underflow issues in the computation, we calculate the logarithmic joint posterior probability $log\mathbb{P(}y_{0},...,y_{n},x_{0},...,x_{n})$ instead:

| $log\mathbb{P(}y_{0},...,y_{n},x_{0},...,x_{n})$ | $=log \left[ \mathbb{P(}y_{0})\prod_{t=1}^{n} \mathbb{P(}y_{t} \vert y_{t-1})\prod_{t=0}^{n} \mathbb{P(}x_{t} \vert y_{t}) \right]$ |
| --- | --- |
|  | $=log\mathbb{P(}y_{0})+\sum_{t=1}^{n} log\mathbb{P(}y_{t} \vert y_{t-1})+\sum_{t=0}^{n} log\mathbb{P(}x_{t} \vert y_{t})$ |
|  | $=log \pi_{y_{0}}+\sum_{t=1}^{n} log \Gamma_{y_{t-1},y_{t}}+\sum_{t=0}^{n} log \Phi_{y_{t}}(x_{t})$ |

Note that notation is introduced in the methods section of the main manuscript (sections “Simulations” and “Sampling from the posterior”).

To compute the log joint posterior probability of the decoded hybrid sequence $s_{0},...,s_{n}$we can compute the following:

$$log\mathbb{P(}y_{0},...,y_{n} =s_{0},...,s_{n},x_{0},...,x_{n}) =log \pi_{y_{0}=s_{0}}+\sum_{t=1}^{n} log \Gamma_{y_{t-1} = s_{t-1},y_{t}=s_{t}}+\sum_{t=0}^{n} log \Phi_{y_{t}=s_{t}}(x_{t})$$

#### Average pointwise posterior probability

##### Pointwise posterior probability

Posterior decoding maximises the pointwise posterior probability $\mathbb{P(}y_{t} | x_{0},...,x_{n})$. This can be rewritten as:

| $\mathbb{P(}y_{t} \vert x_{0},...,x_{n})$ | $=\frac{\mathbb{P(}y_{t},x_{0},...,x_{n})}{\mathbb{P(}x_{0},...,x_{n})}$ |
| --- | --- |
|  | $=\frac{\mathbb{P(}x_{t+1},...,x_{n} \vert y_{t},x_{0},...,x_{t}\mathbb{)P(}y_{t},x_{0},...,x_{t})}{\mathbb{P(}x_{0},...,x_{n})}$ |
|  | $=\frac{\mathbb{P(}x_{t+1},...,x_{n} \vert y_{t}\mathbb{)P(}y_{t},x_{0},...,x_{t})}{\mathbb{P(}x_{0},...,x_{n})}$ |

The probabilities in the numerator are calculated using the forward and backward probabilities.

##### Forward algorithm

For $t=1,...,n$ and all possible values of $y_{t}$, we use the recursion:

|  | $\zeta_{t}(y_{t})\mathbb{=P(}y_{t},x_{0},...,x_{t})$ |
| --- | --- |
|  | $=\sum_{y_{1}}^{y_{t-1}} \mathbb{P(}y_{t},y_{t-1},x_{0},...,x_{t})$ |
|  | $=\sum_{y_{1}}^{y_{t-1}} \left[ \mathbb{P(}x_{t} \vert y_{t}, y_{t-1},x_{0},...,x_{t-1}\mathbb{)P(}y_{t},y_{t-1},x_{0},...,x_{t-1}) \right]$ |
|  | $=\sum_{y_{1}}^{y_{t-1}} \left[ \mathbb{P(}x_{t} \vert y_{t},y_{t-1},x_{0},...,x_{t-1}\mathbb{)P(}y_{t} \vert y_{t-1},x_{0},...,x_{t-1}\mathbb{)P(}y_{t-1},x_{0},...,x_{t-1}) \right]$ |
|  | $=\sum_{y_{1}}^{y_{t-1}} \left[ \mathbb{P(}x_{t} \vert y_{t}\mathbb{)P(}y_{t} \vert y_{t-1}\mathbb{)P(}y_{t-1},x_{0},...,x_{t-1}) \right]$ |
|  | $=\sum_{y_{1}}^{y_{t-1}} \left[ \Phi_{y_{t}}(x_{t})\Gamma_{y_{t-1},y_{t}}\zeta_{t-1}(y_{t-1}) \right]$ |

For all possible values of $y_{0}$, start by defining:

| $\zeta_{0}(y_{0})$ | $\mathbb{=P(}y_{0},x_{0})$ |
| --- | --- |
|  | $\mathbb{=P(}x_{0} \vert y_{0}\mathbb{)P(}y_{0})$ |
|  | $=\Phi_{y_{0}}(x_{0})\pi_{y_{0}}$ |

##### Backward algorithm

For all possible values of $y_{n}$, start by defining $\beta_{n}(y_{n})=1$. For $t=n-1,...,0$ and all possible values of $y_{t}$, we use the recursion:

| $\beta_{t}(y_{t})$ | $\mathbb{=P(}x_{t+1},...,x_{n} \vert y_{t})$ |
| --- | --- |
|  | $=\sum_{y_{t+1}}^{n} \mathbb{P(}x_{t+1},...,x_{n},y_{t+1} \vert y_{t})$ |
|  | $=\sum_{y_{t+1}}^{n} \left[ \mathbb{P(}x_{t+2},...,x_{n} \vert x_{t+1},y_{t+1},y_{t}\mathbb{)P(}x_{t+1},y_{t+1} \vert y_{t}) \right]$ |
|  | $=\sum_{y_{t+1}}^{n} \left[ \mathbb{P(}x_{t+2},...,x_{n} \vert x_{t+1},y_{t+1},y_{t}\mathbb{)P(}x_{t+1} \vert y_{t+1},y_{t}\mathbb{)P(}y_{t+1} \vert y_{t}) \right]$ |
|  | $=\sum_{y_{t+1}}^{n} \left[ \mathbb{P(}x_{t+2},...,x_{n} \vert y_{t+1}\mathbb{)P(}x_{t+1} \vert y_{t+1}\mathbb{)P(}y_{t+1} \vert y_{t}) \right]$ |
|  | $=\sum_{y_{t+1}}^{n} \left[ \beta_{t+1}(y_{t+1})\Phi_{y_{t+1}}(x_{t+1})\Gamma_{y_{t},y_{t+1}} \right]$ |

Using the forward and backward probabilities, the pointwise posterior probability can be written as:

$$\mathbb{P(}y_{t} | x_{0},...,x_{n})=\frac{\beta_{t}(y_{t})\zeta_{t}(y_{t})}{\mathbb{P(}x_{0},...,x_{n})}$$

The likelihood of data $\mathbb{P(}x_{0},...,x_{n})$ can be calculated using the forward probabilities:

| $\mathbb{P(}x_{0},...,x_{n})$ | $=\sum_{y_{0}}^{y_{n}} \mathbb{P(}y_{t},x_{0},...,x_{n})$ |
| --- | --- |
|  | $=\sum_{y_{0}}^{y_{n}} \zeta_{t}(y_{t})$ |

##### Average pointwise posterior probability

Once the forward and backward tables are computed, given a hidden state sequence, we can compute the average pointwise posterior probability as:

$$\frac{1}{m}\sum_{t=0}^{n} \mathbb{P(}y_{t}=s_{t} | x_{0},...,x_{n})=\frac{1}{m}\sum_{t=0}^{n} \frac{\beta_{t}(y_{t} = s_{t})\zeta_{t}(y_{t} = s_{t})}{\sum_{y_{n}} \zeta_{n}(y_{n} = s_{t})}$$

### S5 - Sensitivity and specificity of hybrid decodings in 10 replicates

In the main text, we describe that the optimal 𝛼 value based on sensitivity and specificity is higher than the one obtained with Artemis analysis. To test if this is a spurious result, we replicated the simulation study 10 times (Figure S15, Table S6). We obtained a similar result in which the optimal 𝛼 based on sensitivity is higher than the optimal alpha obtained with Artemis in all replicates.

Thus, even though the statistics that each algorithm maximises for suggest an 𝛼 around 0.75, the user could potentially decide using an 𝛼 of about 0.82, more proximal to the Viterbi solution, to achieve a more balanced result in terms of sensitivity and specificity.


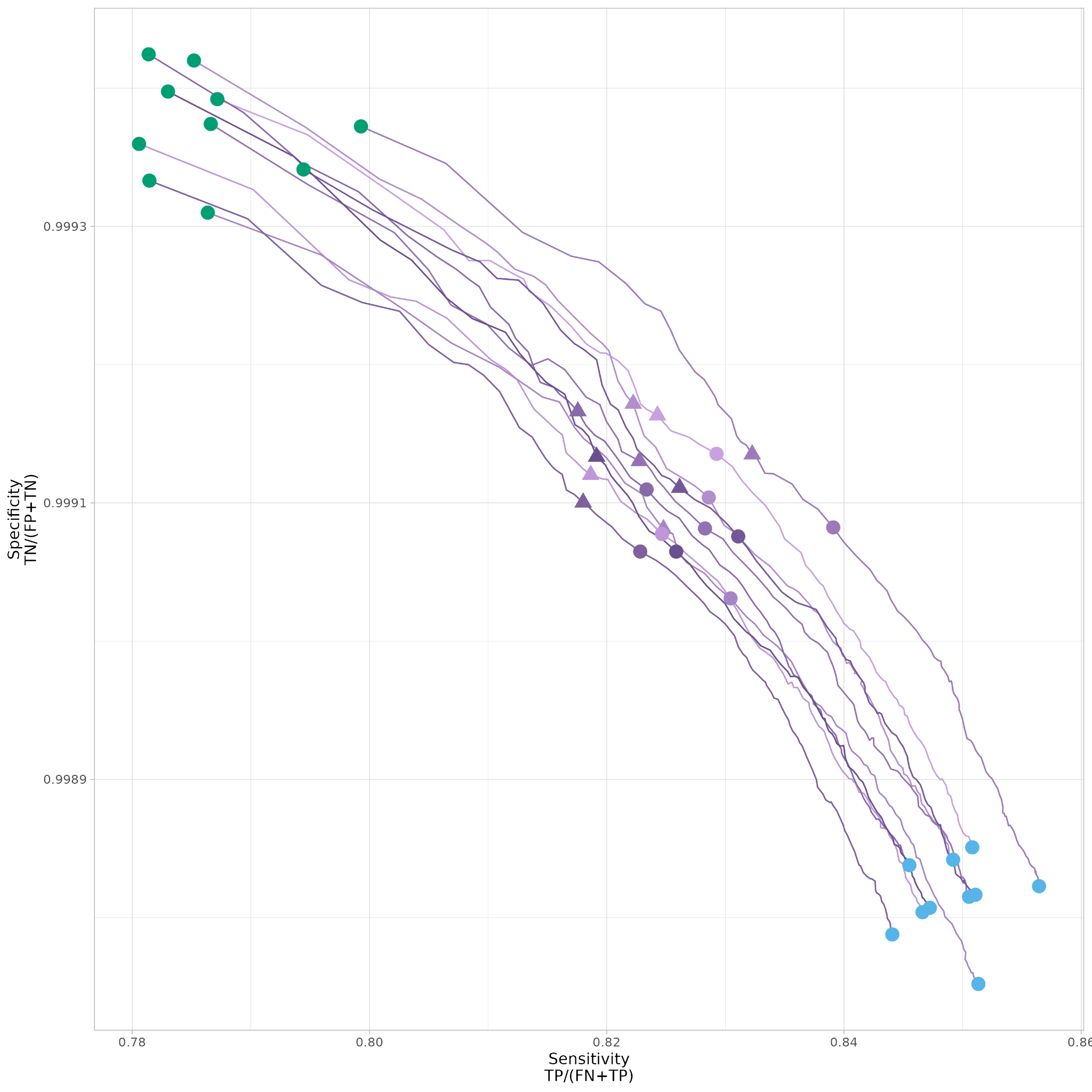


##### Figure S15. Sensitivity (TN/(FP+TN)) and specificity (TP/(FN+TP)) values of Posterior decoding, Viterbi and hybrid decodings based on 10 simulations (shades of purple) of 10^6^ 1kb windows of the trained realistic parameters. Hybrid decoding paths in a grid search for 𝛼 (min value = 0, max value = 1, step size = 0.01), which determine the purple paths. Viterbi and Posterior decoding values are shown as green and blue dots in the extremes, respectively. Optimal 𝛼’s found with the Artemis analysis are shown as dots. Optimal 𝛼’s found based on sensitivity and specificity values are shown as triangles.

##### Table S6. Optimal 𝛼 values based on Artemis analysis or maximising sensitivity and specificity simultaneously for results of hybrid decoding on 10 simulations of the decoded realistic parameters.

| Simulation | Optimal 𝛼 (Artemis) | Optimal 𝛼  (Sensitivity / Specificity) |
| --- | --- | --- |
| 1 | 0.76 | 0.81 |
| 2 | 0.76 | 0.83 |
| 3 | 0.76 | 0.83 |
| 4 | 0.76 | 0.82 |
| 5 | 0.75 | 0.82 |
| 6 | 0.76 | 0.83 |
| 7 | 0.76 | 0.82 |
| 8 | 0.76 | 0.81 |
| 9 | 0.75 | 0.79 |
| 10 | 0.76 | 0.85 |

### S6 - Hybrid decodings compared to Viterbi and Posterior decoding on real data

##### Table S7. Number of archaic fragments that match (overlap for at least a base pair) between decoding methods per individuals’ haplotypes. Rows correspond to the method from which the target fragments have been obtained. Columns correspond to the method by which the fragments are being compared to. For cells with the same method in the row and column, the value indicates the total number of archaic fragments inferred by that method. When the row is different from the column, the value corresponds to the number of fragments of the row method overlapping with the column method. If a cell is subdivided into three cells, the first entry indicates the number of fragments from the row method that overlap with a single fragment with the column method, the second, the overlaps with two fragments and the third entry, with three.

| NA19078 (hap1) | Viterbi | Hybrid | | | Posterior | | |
| --- | --- | --- | --- | --- | --- | --- | --- |
| Viterbi | 636 | 627 | 8 | 1 | 622 | 13 | 1 |
| Hybrid | 646 | 834 | | | 824 | 10 |  |
| Posterior | 651 | 844 | | | 964 | | |

| NA19078 (hap2) | Viterbi | Hybrid | | | Posterior | | |
| --- | --- | --- | --- | --- | --- | --- | --- |
| Viterbi | 633 | 622 | 11 | 0 | 614 | 18 | 1 |
| Hybrid | 644 | 836 | | | 825 | 10 | 1 |
| Posterior | 653 | 848 | | | 972 | | |

| NA20810 (hap1) | Viterbi | Hybrid | | | Posterior | | |
| --- | --- | --- | --- | --- | --- | --- | --- |
| Viterbi | 510 | 498 | 10 | 2 | 490 | 18 | 2 |
| Hybrid | 524 | 684 | | | 674 | 10 | 0 |
| Posterior | 532 | 694 | | | 783 | | |

| NA20810 (hap2) | Viterbi | Hybrid | | | Posterior | | |
| --- | --- | --- | --- | --- | --- | --- | --- |
| Viterbi | 542 | 529 | 13 | 0 | 520 | 20 | 2 |
| Hybrid | 555 | 729 | | | 719 | 9 | 1 |
| Posterior | 566 | 740 | | | 862 | | |

| NA21130 (hap1) | Viterbi | Hybrid | | | Posterior | | |
| --- | --- | --- | --- | --- | --- | --- | --- |
| Viterbi | 550 | 531 | 19 | 0 | 528 | 19 | 3 |
| Hybrid | 569 | 749 | | | 741 | 8 | 0 |
| Posterior | 575 | 757 | | | 884 | | |

| NA21130 (hap2) | Viterbi | Hybrid | | | Posterior | | |
| --- | --- | --- | --- | --- | --- | --- | --- |
| Viterbi | 535 | 519 | 16 | 0 | 515 | 19 | 1 |
| Hybrid | 551 | 729 | | | 719 | 10 | 0 |
| Posterior | 556 | 739 | | | 870 | | |

###

### S7 - Method comparison for fragment edge inference

In this section, we test the precision of the Viterbi, Posterior decoding and hybrid decoding methods to recover the edges of the fragments. Figure S16 shows that all three methods are not biased in calling upstream or downstream, nor the start nor the end of the fragments. However, the absolute difference between true and inferred edges is of 6 to 7kb (6-7 windows) on average.

#####
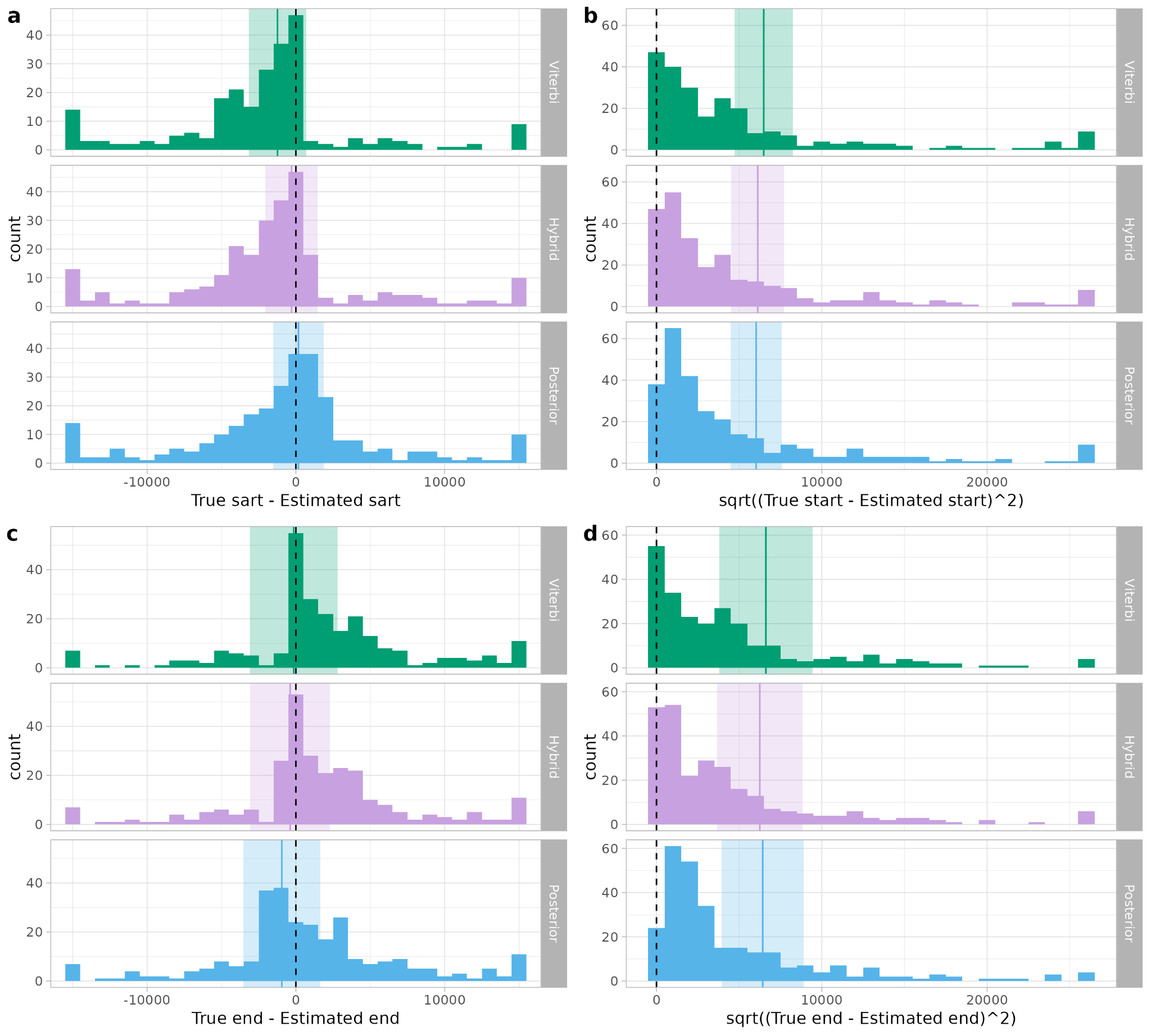


##### Figure S16. Histogram of the difference between the true edge of the fragment and the inferred edge. True simulated fragments correspond to realistic parameter simulation. Hybrid decoding run with 𝛂 = 0.75 (optimal alpha in main text). a) and b) correspond to the start of the fragment. c) and d) correspond to the end of the fragment. a) and c) are the true edge - estimated edge; negative values correspond to the estimated value being downstream from the true starting value, and positive values upstream. b) and d) the difference is expressed with absolute values. Vertical dotted black lines denote the 0 value.

### S8 - Linkage Disequilibrium

##### Table S8. Linkage disequilibrium measured as $r^{2}$ between all pairs of observations in the chr1:159203000-159329000 haplotype 1 of individual NA19078. All SNPs are also found in Neanderthal samples, except for SNP 159305198. SNPs identified in the second fragment called by Posterior decoding in Figure 8 are colored in grey.

| CHR | POS1 | POS2 | N_INDV | R^2 |
| --- | --- | --- | --- | --- |
| chr1 | 159223610 | 159229803 | 726 | 0.98749 |
| chr1 | 159223610 | 159234010 | 726 | 0.951575 |
| chr1 | 159223610 | 159237763 | 726 | 0.98749 |
| chr1 | 159223610 | 159266590 | 726 | 0.775764 |
| chr1 | 159223610 | 159268150 | 726 | 0.775764 |
| chr1 | 159223610 | 159279268 | 726 | 0.775764 |
| chr1 | 159223610 | 159295573 | 726 | 0.421865 |
| chr1 | 159223610 | 159298774 | 726 | 0.412193 |
| chr1 | 159223610 | 159304153 | 726 | 0.377119 |
| chr1 | 159223610 | 159305198 | 726 | 0.367855 |
| chr1 | 159223610 | 159307210 | 726 | 0.377119 |
| chr1 | 159229803 | 159234010 | 726 | 0.939522 |
| chr1 | 159229803 | 159237763 | 726 | 0.975098 |
| chr1 | 159229803 | 159266590 | 726 | 0.765863 |
| chr1 | 159229803 | 159268150 | 726 | 0.765863 |
| chr1 | 159229803 | 159279268 | 726 | 0.765863 |
| chr1 | 159229803 | 159295573 | 726 | 0.415617 |
| chr1 | 159229803 | 159298774 | 726 | 0.406075 |
| chr1 | 159229803 | 159304153 | 726 | 0.371409 |
| chr1 | 159229803 | 159305198 | 726 | 0.362271 |
| chr1 | 159229803 | 159307210 | 726 | 0.371409 |
| chr1 | 159234010 | 159237763 | 726 | 0.963625 |
| chr1 | 159234010 | 159266590 | 726 | 0.760736 |
| chr1 | 159234010 | 159268150 | 726 | 0.760736 |
| chr1 | 159234010 | 159279268 | 726 | 0.760736 |
| chr1 | 159234010 | 159295573 | 726 | 0.397687 |
| chr1 | 159234010 | 159298774 | 726 | 0.388518 |
| chr1 | 159234010 | 159304153 | 726 | 0.355023 |
| chr1 | 159234010 | 159305198 | 726 | 0.346247 |
| chr1 | 159234010 | 159307210 | 726 | 0.355023 |
| chr1 | 159237763 | 159266590 | 726 | 0.78994 |
| chr1 | 159237763 | 159268150 | 726 | 0.78994 |
| chr1 | 159237763 | 159279268 | 726 | 0.78994 |
| chr1 | 159237763 | 159295573 | 726 | 0.415617 |
| chr1 | 159237763 | 159298774 | 726 | 0.406075 |
| chr1 | 159237763 | 159304153 | 726 | 0.371409 |
| chr1 | 159237763 | 159305198 | 726 | 0.362271 |
| chr1 | 159237763 | 159307210 | 726 | 0.371409 |
| chr1 | 159266590 | 159268150 | 726 | 1 |
| chr1 | 159266590 | 159279268 | 726 | 1 |
| chr1 | 159266590 | 159295573 | 726 | 0.505722 |
| chr1 | 159266590 | 159298774 | 726 | 0.494044 |
| chr1 | 159266590 | 159304153 | 726 | 0.468551 |
| chr1 | 159266590 | 159305198 | 726 | 0.457215 |
| chr1 | 159266590 | 159307210 | 726 | 0.468551 |
| chr1 | 159268150 | 159279268 | 726 | 1 |
| chr1 | 159268150 | 159295573 | 726 | 0.505722 |
| chr1 | 159268150 | 159298774 | 726 | 0.494044 |
| chr1 | 159268150 | 159304153 | 726 | 0.468551 |
| chr1 | 159268150 | 159305198 | 726 | 0.457215 |
| chr1 | 159268150 | 159307210 | 726 | 0.468551 |
| chr1 | 159279268 | 159295573 | 726 | 0.505722 |
| chr1 | 159279268 | 159298774 | 726 | 0.494044 |
| chr1 | 159279268 | 159304153 | 726 | 0.468551 |
| chr1 | 159279268 | 159305198 | 726 | 0.457215 |
| chr1 | 159279268 | 159307210 | 4091 | 0.549613 |
| chr1 | 159295573 | 159298774 | 4091 | 0.954857 |
| chr1 | 159295573 | 159304153 | 4091 | 0.955431 |
| chr1 | 159295573 | 159305198 | 4091 | 0.891118 |
| chr1 | 159295573 | 159307210 | 4091 | 0.955431 |
| chr1 | 159298774 | 159304153 | 4091 | 0.910658 |
| chr1 | 159298774 | 159305198 | 4091 | 0.933327 |
| chr1 | 159298774 | 159307210 | 4091 | 0.910658 |
| chr1 | 159304153 | 159305198 | 4091 | 0.935943 |
| chr1 | 159304153 | 159307210 | 4091 | 1 |
| chr1 | 159305198 | 159307210 | 4091 | 0.935943 |

## Bibliography

1. [Adrion JR, Cole CB, Dukler N, Galloway JG, Gladstein AL, Gower G, Kyriazis CC, Ragsdale AP, Tsambos G, Baumdicker F, et al. 2020. A community-maintained standard library of population genetic models. eLife 9:e54967.](https://app.readcube.com/library/?style=Molecular%20Biology%20and%20Evolution+%7B%22language%22:%22en-US%22%7D)
2. [Bæk ZED, Coll Macià M, Skov L, Hobolth A. 2025. Advanced posterior analyses of hidden Markov models: finite Markov chain imbedding and hybrid decoding. arXiv.](https://app.readcube.com/library/?style=Molecular%20Biology%20and%20Evolution+%7B%22language%22:%22en-US%22%7D)
3. [Baumdicker F, Bisschop G, Goldstein D, Gower G, Ragsdale AP, Tsambos G, Zhu S, Eldon B, Ellerman EC, Galloway JG, et al. 2021. Efficient ancestry and mutation simulation with msprime 1.0. Genetics 220:iyab229.](https://app.readcube.com/library/?style=Molecular%20Biology%20and%20Evolution+%7B%22language%22:%22en-US%22%7D)
4. [Jacobs GS, Hudjashov G, Saag L, Kusuma P, Darusallam CC, Lawson DJ, Mondal M, Pagani L, Ricaut F-X, Stoneking M, et al. 2019. Multiple Deeply Divergent Denisovan Ancestries in Papuans. Cell.](https://app.readcube.com/library/?style=Molecular%20Biology%20and%20Evolution+%7B%22language%22:%22en-US%22%7D)
5. [Lauterbur ME, Cavassim MIA, Gladstein AL, Gower G, Pope NS, Tsambos G, Adrion J, Belsare S, Biddanda A, Caudill V, et al. 2023. Expanding the stdpopsim species catalog, and lessons learned for realistic genome simulations. eLife 12:RP84874.](https://app.readcube.com/library/?style=Molecular%20Biology%20and%20Evolution+%7B%22language%22:%22en-US%22%7D)
6. [Skov L, Hui R, Shchur V, Hobolth A, Scally A, Schierup MH, Durbin R. 2018. Detecting archaic introgression using an unadmixed outgroup. Plos Genet 14:e1007641.](https://app.readcube.com/library/?style=Molecular%20Biology%20and%20Evolution+%7B%22language%22:%22en-US%22%7D)
7. [Skov L, Coll Macià M, Sveinbjörnsson G, Mafessoni F, Lucotte EA, Einarsdóttir MS, Jonsson H, Halldorsson B, Gudbjartsson DF, Helgason A, et al. 2020. The nature of Neanderthal introgression revealed by 27,566 Icelandic genomes. Nature:1–6.](https://app.readcube.com/library/?style=Molecular%20Biology%20and%20Evolution+%7B%22language%22:%22en-US%22%7D)
8. [Adrion JR, Cole CB, Dukler N, Galloway JG, Gladstein AL, Gower G, Kyriazis CC, Ragsdale AP, Tsambos G, Baumdicker F, et al. 2020. A community-maintained standard library of population genetic models. eLife 9:e54967.](https://app.readcube.com/library/?style=Molecular%20Biology%20and%20Evolution+%7B%22language%22:%22en-US%22%7D)
9. [Bæk ZED, Coll Macià M, Skov L, Hobolth A. 2025. Advanced posterior analyses of hidden Markov models: finite Markov chain imbedding and hybrid decoding. arXiv.](https://app.readcube.com/library/?style=Molecular%20Biology%20and%20Evolution+%7B%22language%22:%22en-US%22%7D)
10. [Baumdicker F, Bisschop G, Goldstein D, Gower G, Ragsdale AP, Tsambos G, Zhu S, Eldon B, Ellerman EC, Galloway JG, et al. 2021. Efficient ancestry and mutation simulation with msprime 1.0. Genetics 220:iyab229.](https://app.readcube.com/library/?style=Molecular%20Biology%20and%20Evolution+%7B%22language%22:%22en-US%22%7D)
11. [Jacobs GS, Hudjashov G, Saag L, Kusuma P, Darusallam CC, Lawson DJ, Mondal M, Pagani L, Ricaut F-X, Stoneking M, et al. 2019. Multiple Deeply Divergent Denisovan Ancestries in Papuans. Cell.](https://app.readcube.com/library/?style=Molecular%20Biology%20and%20Evolution+%7B%22language%22:%22en-US%22%7D)
12. [Lauterbur ME, Cavassim MIA, Gladstein AL, Gower G, Pope NS, Tsambos G, Adrion J, Belsare S, Biddanda A, Caudill V, et al. 2023. Expanding the stdpopsim species catalog, and lessons learned for realistic genome simulations. eLife 12:RP84874.](https://app.readcube.com/library/?style=Molecular%20Biology%20and%20Evolution+%7B%22language%22:%22en-US%22%7D)
13. [Skov L, Hui R, Shchur V, Hobolth A, Scally A, Schierup MH, Durbin R. 2018. Detecting archaic introgression using an unadmixed outgroup. Plos Genet 14:e1007641.](https://app.readcube.com/library/?style=Molecular%20Biology%20and%20Evolution+%7B%22language%22:%22en-US%22%7D)
14. [Skov L, Coll Macià M, Sveinbjörnsson G, Mafessoni F, Lucotte EA, Einarsdóttir MS, Jonsson H, Halldorsson B, Gudbjartsson DF, Helgason A, et al. 2020. The nature of Neanderthal introgression revealed by 27,566 Icelandic genomes. Nature:1–6.](https://app.readcube.com/library/?style=Molecular%20Biology%20and%20Evolution+%7B%22language%22:%22en-US%22%7D)
